# K29‐Linked Ubiquitination of Transcription Regulators Controls Cell Proliferation in the Unfolded Protein Response

**DOI:** 10.1002/advs.202509817

**Published:** 2025-08-30

**Authors:** Qiushuang Zhang, Xucong Teng, Yicong Dai, Yuncong Wu, Hongwei Hou, Jinghong Li

**Affiliations:** ^1^ Beijing Life Science Academy Beijing 102209 China; ^2^ New Cornerstone Science Laboratory Department of Chemistry Key Laboratory of Bioorganic Phosphorus Chemistry & Chemical Biology Tsinghua University Beijing 100084 China; ^3^ Center for Bioanalytical Chemistry Hefei National Laboratory of Physical Science at Microscale University of Science and Technology of China Hefei 230026 China

**Keywords:** cohesin complex, K29‐linked ubiquitination, transcription regulation, unfolded protein response

## Abstract

The ubiquitin chains perform diverse biological functions through different linkages. However, the understanding of non‐canonical K29‐linked ubiquitin chains is relatively limited. Exploring the physiological functions of K29‐linked ubiquitin chains beyond degradation is crucial for deciphering the ubiquitin chain code, which is essential for understanding cellular physiology. The unfolded protein response (UPR) serves as a crucial mechanism for cells to cope with endoplasmic reticulum stress and involves comprehensive and precise regulation. Ubiquitin, as a regulator of protein function, has potential regulatory functions other than guiding protein degradation in the UPR. Here, a close association is revealed between K29‐linked ubiquitin chains and transcriptional regulation during the UPR. After UPR induction, the K29‐linked ubiquitination of the SMC1A and SMC3 proteins in the cohesin complex increases. The transcription of cell proliferation‐related genes, such as SERTAD1 and NUDT16L1, is regulated by the K29‐linked ubiquitination of cohesin. Overall, the upregulation of K29‐linked ubiquitination of cohesin during the UPR disrupts the formation of the transcription initiation complex, resulting in the transcriptional downregulation of cell proliferation‐related genes.

## Graphical abstract

Under endoplasmic reticulum stress, cells activate the unfolded protein response (UPR). During the UPR, the cohesin complex at the promoter of cell proliferation‐related genes undergoes K29‐linked ubiquitination, recruiting the cohesin release factor WAPL to release cohesin, leading to transcriptional downregulation and inhibition of cell growth.

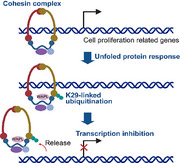

## 1 Introduction

Ubiquitin (Ub) is a 76‐amino acid molecule^[^ ^1^ ^]^ that primarily modifies lysine residues^[^ ^2^ ^]^ on protein substrates through its C‐terminal carboxyl group. Ubiquitination is an essential post‐translational modification (PTM) formed through an enzymatic cascade of activating enzymes (E1), conjugating enzymes (E2), and ligases (E3).^[^ ^3^ ^]^ In addition to modifying substrates with monomers (monoubiquitination), ubiquitin can also form polymers.^[^ ^4^ ^]^ The preceding ubiquitin can conjugate to the subsequent ubiquitin through one of its seven lysine (K) residues or its N‐terminus; thus, there are eight types of linkages: M1‐, K6‐, K11‐, K27‐, K29‐, K33‐, K48‐, and K63‐linked linkages.^[^ ^5^ ^]^ Among these, K48‐linked and K63‐linked ubiquitin chains are the most abundant ubiquitin chains involved in cellular processes such as protein degradation by the ubiquitin‒proteasome system (UPS),^[^ ^6^ ^]^ lysosomal autophagy,^[^ ^7^ ^]^ DNA damage repair,^[^ ^8^ ^]^ and cell cycle regulation.^[^ ^9^ ^]^ The K29‐linked ubiquitin chain, a non‐canonical ubiquitin chain whose abundance follows only K48‐linked chains, has been shown to participate in physiological processes, including protein degradation,^[^ ^10^, ^11^ ^]^ viral infection,^[^ ^12^ ^]^ and cell cycle regulation.^[^ ^13^ ^]^ However, our knowledge of K29‐linked ubiquitin chains is relatively limited compared with that of other types of ubiquitin chains,^[^ ^13^, ^14^ ^]^ and further exploration is needed to fully understand their functions.

The cellular stress response is a crucial mechanism by which cells maintain homeostasis under external stimuli. When unfolded or misfolded proteins accumulate in the endoplasmic reticulum, cells activate the unfolded protein response (UPR) to ensure cell survival. Research indicates that activation of the UPR pathway is closely associated with various human diseases,^[^ ^15^ ^]^ including cancer,^[^ ^16^, ^17^, ^18^ ^]^ diabetes,^[^ ^19^ ^]^ neurodegenerative diseases,^[^ ^20^, ^21^, ^22^, ^23^ ^]^ and more.^[^ ^24^, ^25^, ^26^, ^27^, ^28^ ^]^ Understanding the detailed regulatory mechanisms of the UPR is essential for developing novel therapeutic strategies for these diseases.^[^ ^15^ ^]^ The UPR consists of a series of signaling pathways primarily mediated by three transmembrane proteins that sense ER stress and initiate downstream regulation: inositol‐requiring enzyme 1 (IRE1/ERN1), activating transcription factor 6 (ATF6), and protein kinase RNA‐like ER kinase (PERK, EIF2AK3).^[^ ^29^ ^]^ These pathways involve comprehensive and precise regulation of gene transcription, RNA translation, and protein modification. During this process, cells need to reduce overall mRNA translation levels to decrease protein load; selectively activate gene expression related to amino acid metabolism, autophagy, and protein folding; and utilize ER‐associated protein degradation (ERAD) pathways to clear unfolded or misfolded proteins. Ubiquitin, as a regulatory switch for protein function and stability, has been shown to play a crucial role in the UPR.^[^ ^30^, ^31^, ^32^ ^]^ For example, in ERAD pathways, proteins are modified by K48‐linked or K11‐linked ubiquitin chains, leading to their degradation.^[^ ^33^ ^]^ In addition to guiding protein degradation, ubiquitin modification can promote protein aggregation to drive ER membrane remodeling and ER autophagy.^[^ ^34^ ^]^ Notably, the activation of the UPR can halt cell proliferation, allowing cells to concentrate materials and energy to recover from ER stress.^[^ ^35^ ^]^ Numerous proteins, such as transcription factors and enzymes, are involved in comprehensive and precise transcriptional regulation during this process. These proteins are potential substrates for ubiquitination. The role of ubiquitin chain modifications, especially the less‐studied K29‐linked ubiquitin chain, in transcriptional regulation during the UPR process and its specific regulatory mechanisms remain to be investigated.

Here, we report a new transcriptional regulatory function of the K29‐linked ubiquitin chain in the UPR. During the UPR, there is a significant upregulation of K29‐linked ubiquitin chains on the cohesin complex, with the K1222 site on SMC1A potentially being a ubiquitination site. The genes SERTAD1 and NUDT16L1, which are associated with cell proliferation, are target genes regulated by K29‐linked ubiquitination of the cohesin complex. Overall, we confirmed that the K29‐linked ubiquitination of the cohesin complex regulates the transcription of cell proliferation‐related genes in the UPR.

## 2 Results

### 2.1 K29‐Linked Ubiquitin Chains Are Highly Enriched on Chromatin And Overlap Significantly with Transcriptionally Active Histone Modifications

**Figure 1:**
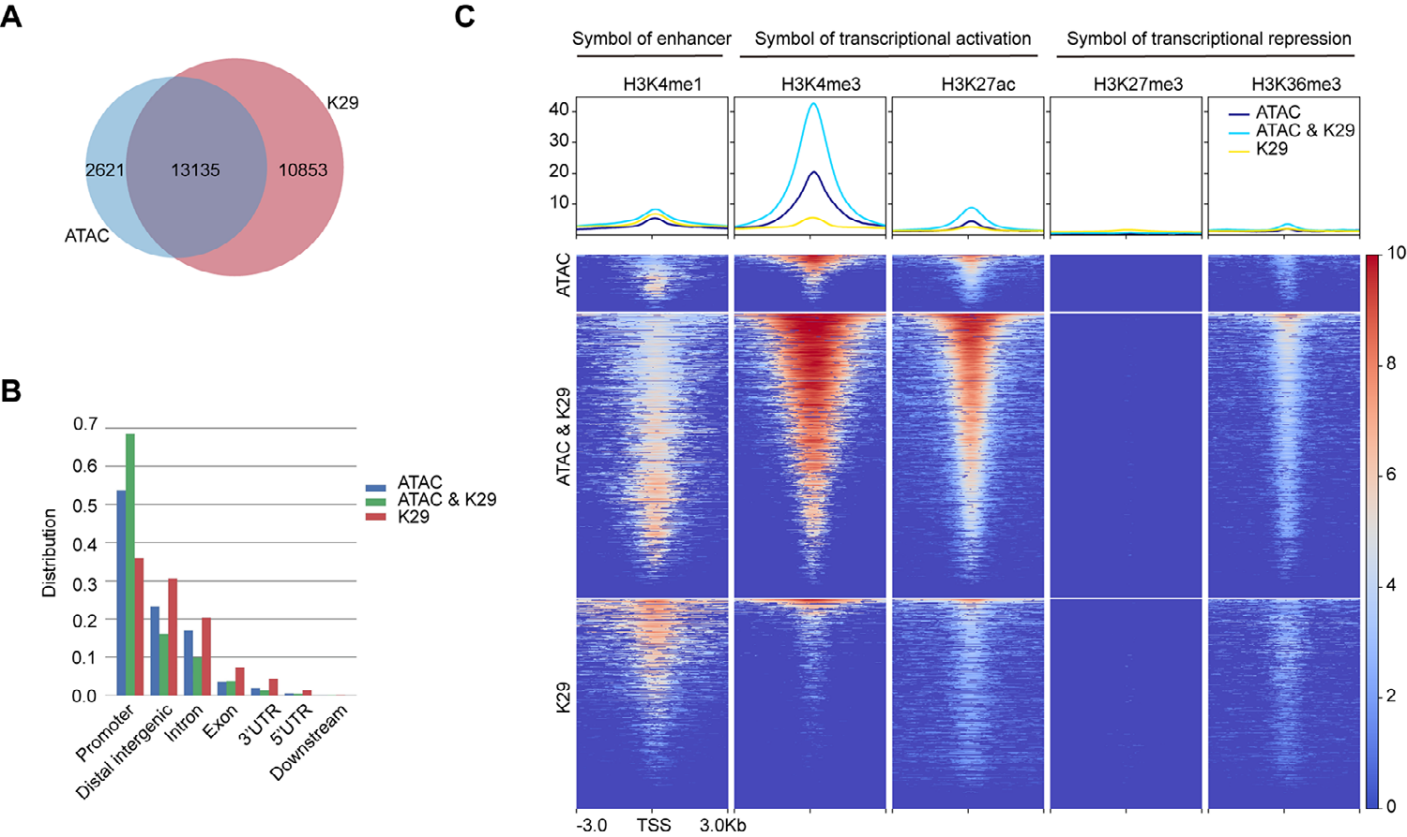
K29‐linked ubiquitin chains are closely related to transcriptional regulation. A) Overlap of ATAC‐seq peaks and K29 CUT&Tag peaks. B) Distribution of peaks at different functional regions in chromatin. C) H3K4me1, H3K4me3, H3K27ac, H3K27me3, and H3K36me3 CUT&Tag density heatmaps for peaks classified as ATAC‐seq only, ATAC‐seq and K29 CUT&Tag, or K29 CUT&Tag only.

To investigate the potential association of K29‐linked ubiquitin chains with epigenetic regulation, we initially explored whether K29‐linked ubiquitin chains are enriched at specific regulatory regions on chromatin. By using Cleavage Under Targets and Tagmentation (CUT&Tag),^[^ ^36^ ^]^ we profiled the chromatin landscape of K29‐linked ubiquitin chains in HEK293FT cells and compared it with five crucial histone modification landscapes, including enhancer marks (H3K4me1), transcription activation marks (H3K4me3 and H3K27ac), and transcription repression marks (H3K27me3 and H3K36me3). For the CUT&Tag analysis of K29‐linked ubiquitin chains, we employed sAB‐K29, as reported by the Liu laboratory,^[^ ^13^ ^]^ which is known for its high specificity toward K29‐linked ubiquitin chains compared with seven other types of ubiquitin chain linkages (Figure S1, Supporting Information). Additionally, we performed an assay for transposase‐accessible chromatin with high‐throughput sequencing (ATAC‐seq)^[^ ^37^ ^]^ to assess chromatin accessibility in HEK293FT cells and conducted integrated analysis with the chromatin landscape of K29‐linked ubiquitin chains. The results indicate a significant overlap between the K29 peaks and ATAC peaks (**Figure** **1**A), with these overlapping peaks notably enriched in promoter regions (Figure 1B). Furthermore, we categorized the peaks with ATAC‐seq and K29 CUT&Tag signals into three classes: peaks with only ATAC‐seq signals, peaks with both ATAC‐seq and K29 CUT&Tag signals, and peaks with only K29 CUT&Tag signals. We then compared these categories with the distribution of five histone modifications on chromatin. We observed that the peaks of K29‐linked ubiquitin chains on chromatin were enriched in the enhancer marker H3K4me1 and strongly enriched in the transcriptional activation markers H3K4me3 and H3K27ac. Surprisingly, the enrichment levels of H3K4me3 and H3K27ac at peaks with both ATAC‐seq and K29 CUT&Tag signals were nearly double those at sites with only ATAC‐seq signals (Figure 1C; Figure S2, Supporting Information). These findings suggest a close association between K29‐linked ubiquitin chains in chromatin modeling and transcriptional regulation.

### 2.2 The Number Of Nuclear And Chromatin K29‐Linked Ubiquitin Chains Decreases During The UPR

Ubiquitination modification plays a crucial role in responding to various stress conditions such as UPR, heat shock, oxidative stress, osmotic stress, UV stress, and proteasome inhibition.^[^ ^38^ ^]^ Notably, under these stress conditions, cells can adapt to environmental changes by temporarily halting processes such as RNA translation and cell proliferation. UPR is an important mechanism by which cells respond to external stress, involving comprehensive and precise transcriptional regulation. Following the discovery of the close relationship between K29‐linked ubiquitination and chromatin transcriptional regulation, we speculated that K29‐linked ubiquitination may be involved in the transcriptional repression regulation of the stress response process. Therefore, we subsequently explored the participation of K29‐linked ubiquitin chains during the UPR. Tunicamycin (Tm) can induce the UPR by inhibiting N‐linked glycosylation, whereas thapsigargin (Tg) can induce the UPR by inhibiting Ca^2+^ ATPase. We treated HEK293FT cells with 2 µg mL^−1^ Tm or 1 µg mL^−1^ Tg for 24 h to establish a chemically induced UPR model. To validate the successful induction of the UPR, we analyzed gene expression changes during the UPR via RNA‐seq. Gene Ontology (GO) analysis of the RNA‐seq data (Figures S3 and S4, Supporting Information) revealed that, compared with those in untreated cells, genes upregulated in chemically induced cells were significantly enriched in endoplasmic reticulum stress‐related pathways, whereas downregulated genes were significantly enriched in cell proliferation‐related pathways, indicating the successful induction of the UPR.

**Figure 2:**
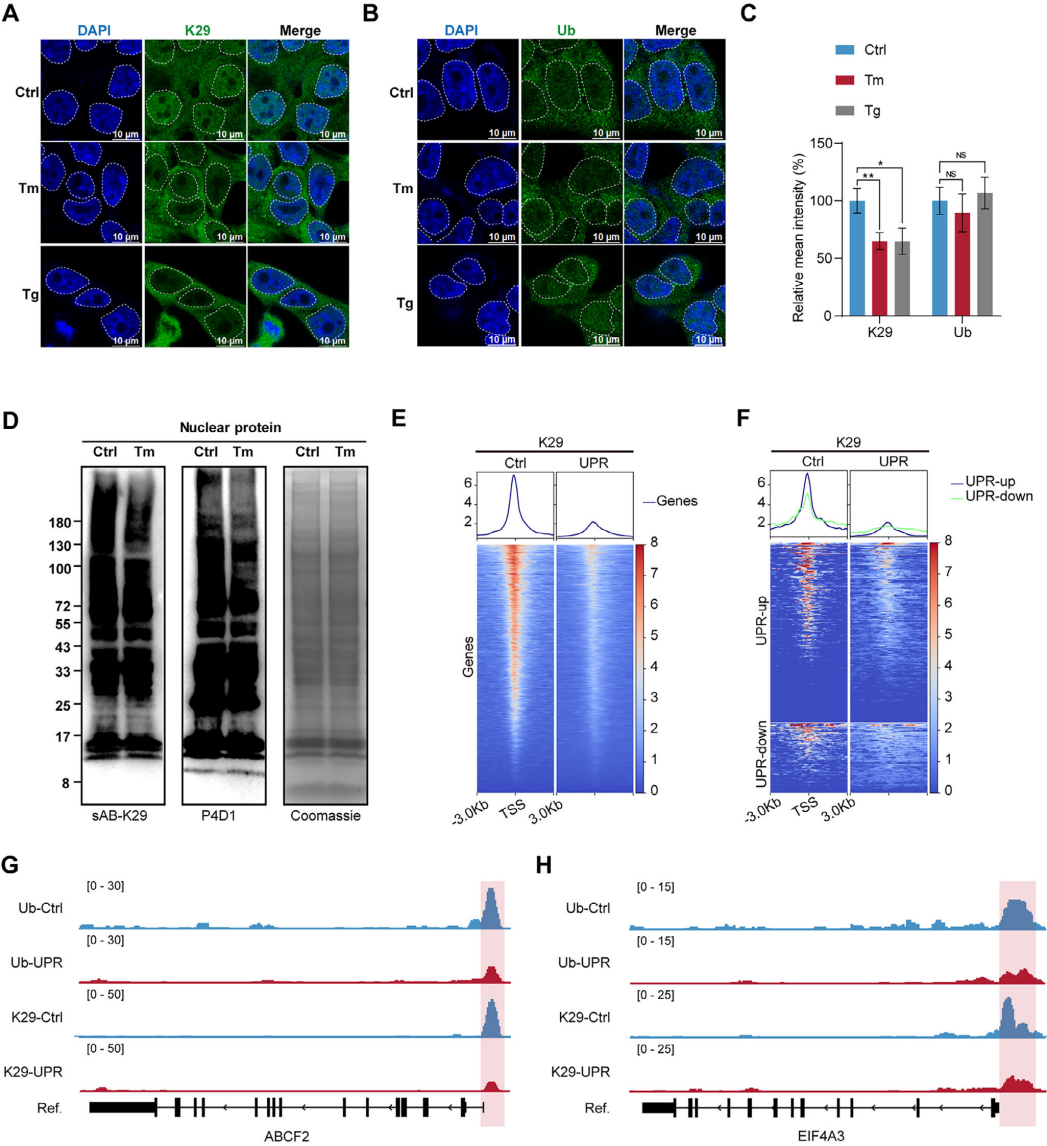
K29‐linked ubiquitin chains are involved in the transcriptional downregulation of genes related to the UPR. Representative immunofluorescence images showing the distribution of A) K29‐linked ubiquitin chains and B) total ubiquitin in HEK293FT cells under induced (Tm, Tg) or untreated (Ctrl) conditions. The nuclei are outlined by white dashed lines. The scale bar represents 10 µm.C) Fluorescence intensity data are presented as the means ± s.d.s (*n* = 100 cells; two‐sided Student's *t*‐test; ns, *p* > 0.05; ^*^, *p* < 0.05; ^**^, *p* < 0.01). D) Coomassie blue‐stained gel of nuclear proteins (right panel) under UPR‐induced (Tm) or untreated (Ctrl) conditions and parallel western blot analysis with sAB‐K29 antibody (left panel) or anti‐monoubiquitin (P4D1) (middle panel) as the primary antibody. E) K29 CUT&Tag density heatmaps across all the genes under induced (UPR) or untreated (Ctrl) conditions. F) K29 CUT&Tag density heatmaps for peaks classified as unregulated genes in the UPR (UPR‐up) or downregulated genes in the UPR (UPR‐down) under induced (UPR) or untreated (Ctrl) conditions. G) and h) CUT&Tag peaks of K29‐linked ubiquitin chains and monoubiquitin around the ABCF2 and ELF4A3 gene loci. The red box indicates the promoter region.

To understand the role of ubiquitin chains in the UPR, determining the changes in the content and distribution of ubiquitin chains in cells before and after UPR induction is essential. Therefore, we performed immunofluorescence imaging using specific antibodies against K27‐, K29‐ (sAB‐K29), K48‐, and K63‐linked ubiquitin chains and monoubiquitin (Figure S5, Supporting Information). Owing to the lack of reliable antibodies, other ubiquitin chains were not detected in this work. We found that after UPR induction with Tm or Tg, the levels of K27‐, K48‐, and K63‐linked ubiquitin chains and total ubiquitin in the nucleus did not significantly change, whereas the number of K29‐linked ubiquitin chains significantly decreased (**Figure** **2**A–C; Figure S5, Supporting Information). Western blotting of nuclear proteins also confirmed the downregulation of K29‐linked ubiquitination in the nucleus (Figure 2D; Figure S15, Supporting Information). These results suggest that among the various types of ubiquitin chains, K29‐linked ubiquitin chains may play a unique and crucial role in the UPR.

Furthermore, to investigate the changes in the chromatin distribution of K29‐linked ubiquitin chains during the UPR, we performed CUT&Tag analysis targeting monoubiquitin and K29‐linked ubiquitin chains in UPR‐induced cells. Compared with that in untreated cells, the enrichment of K29‐linked ubiquitin chains at chromatin promoter sites significantly decreased (Figure 2E). In particular, for genes whose expression is transcriptionally downregulated during the UPR, a notable absence of K29‐linked ubiquitination at promoter regions is observed (Figure 2F). For example, ATP‐binding cassette subfamily F member 2 (ABCF2) is associated with the transport of intracellular substances, while eukaryotic translation initiation factor 4A3 (EIF4A3) is an ATP‐dependent RNA helicase; the transcription levels of these proteins are downregulated in the UPR (Figure S6, Supporting Information). Moreover, they presented a significant loss of K29‐linked ubiquitin chains in the promoter region (Figure 2G,H). These results suggest a potential regulatory role of K29‐linked ubiquitin chains in the transcriptional downregulation of genes related to the UPR.

### 2.3 The Cohesin Complex Is A Substrate of K29‐Linked Ubiquitination During The UPR

Next, we need to identify the substrate for K29‐linked ubiquitination to reveal its regulatory function. Transcription regulators are part of the transcription initiation complex and play a significant role in transcriptional regulation. We hypothesized that certain transcription regulators may undergo K29‐linked ubiquitination during the UPR, thereby mediating the transcriptional downregulation of related genes.

To validate this hypothesis, we collected genes whose expression is transcriptionally downregulated during the UPR induced by Tm. On the basis of epigenetic landscape in silico deletion analysis (LISA),^[^ ^39^ ^]^ we predicted transcription factors that could regulate these genes. Additionally, we identified genes with a decreased distribution of K29‐linked ubiquitin chains at promoter loci and predicted related transcription factors via LISA. By taking the intersection of these two sets, we identified 37 potential regulatory factors (Table S1, Supporting Information).

**Figure 3:**
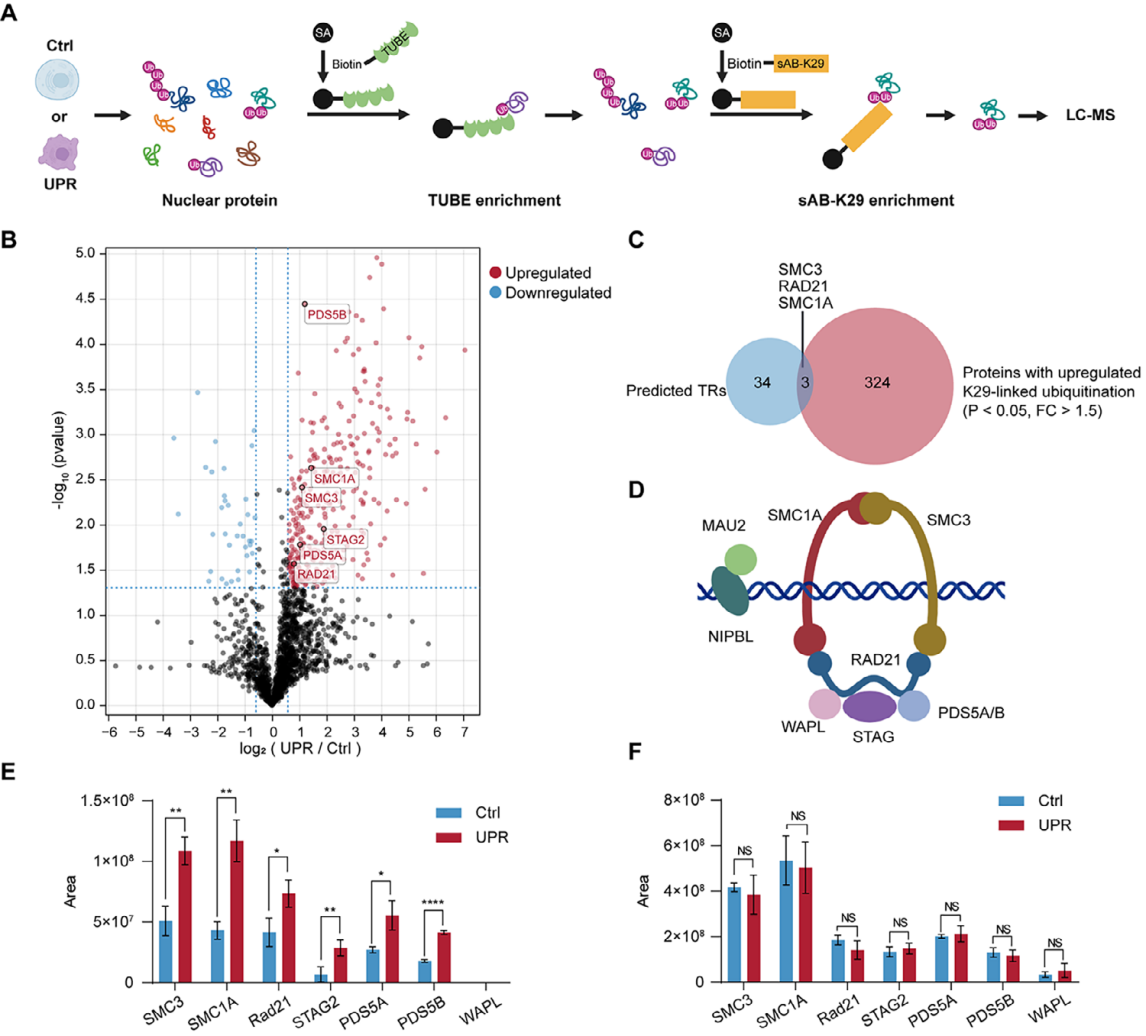
The cohesin complex is a substrate of K29‐linked ubiquitination during the UPR. A) MS workflow for the analysis of K29‐linked ubiquitinated proteins from nuclear proteins. B) Volcano plot of the quantitative mass spectrometry results. Significantly upregulated proteins (fold change > 1.5, *p*‐value < 0.05) are colored red, whereas significantly downregulated proteins (fold change > 1.5, *p‐value* < 0.05) are colored blue. C) Overlap of predicted transcription factors and proteins with upregulated K29‐linked ubiquitination according to mass spectrometry data analysis. D) Schematic diagram of the cohesin complex. E) Peak intensity of cohesin subunits (SMC3, SMC1A, RAD21, STAG2, PDS5A, PDS5B) and the cohesin release factor WAPL after enrichment from the label‐free quantitative mass spectrometry analysis. F) Peak intensity of related proteins before enrichment. (Data are presented as the means ± s.d.s, *n* = 3, two‐sided Student's *t*‐test, ns *p* > 0.05, ^*^ *p* < 0.05, ^**^ *p* < 0.01, ^***^ *p* < 0.001, ^****^ *p* < 0.0001).

To identify which transcription factors undergo K29‐linked ubiquitination, we extracted nuclear proteins from untreated and UPR‐induced cells and then performed sequential affinity enrichment using biotinylated tandem ubiquitin binding entities (TUBEs)^[^ ^40^ ^]^ and sAB‐K29, followed by label‐free quantitative mass spectrometry analysis of the enriched proteins (**Figure** **3**A). The mass spectrometry results revealed that during the UPR, the enrichment of 327 proteins increased, whereas that of 36 proteins decreased (Figure 3B). By comparing the proteins with altered enrichment levels to the 37 predicted regulatory factors, we identified an intersection that included three proteins: SMC3, SMC1A, and RAD21 (Figure 3C). These three proteins are the core subunits of the cohesin complex. Cohesin is a ring‐shaped complex composed of multiple protein subunits (Figure 3D) that mainly participates in the cohesion and separation of sister chromatids after DNA replication.^[^ ^41^ ^]^ Additionally, it is involved in chromatin compaction^[^ ^42^ ^]^ and DNA damage repair^[^ ^43^ ^]^ and can facilitate transcription.^[^ ^44^ ^]^ In addition to SMC3, SMC1A, and RAD21, other subunits, such as STAG2, PDS5A, and PDS5B, were also more enriched under UPR‐induced conditions (Figure 3E). Considering that increased protein expression levels could contribute to this observation, we conducted label‐free quantitative mass spectrometry analysis of total nuclear proteins from induced and untreated cells. Surprisingly, we found no significant changes in the abundance of these subunits of cohesin within the nucleus (Figure 3F), which was validated by the western blotting results (Figure S7a, Supporting Information). These findings suggest an increase in K29‐linked ubiquitination of the cohesin complex when the UPR is induced in cells. Notably, this overall increase in K29‐linked ubiquitination may be attributed to the strong interactions among these subunits, where an increase in K29‐linked ubiquitination of one subunit could lead to the collective enrichment of related proteins.

**Figure 4:**
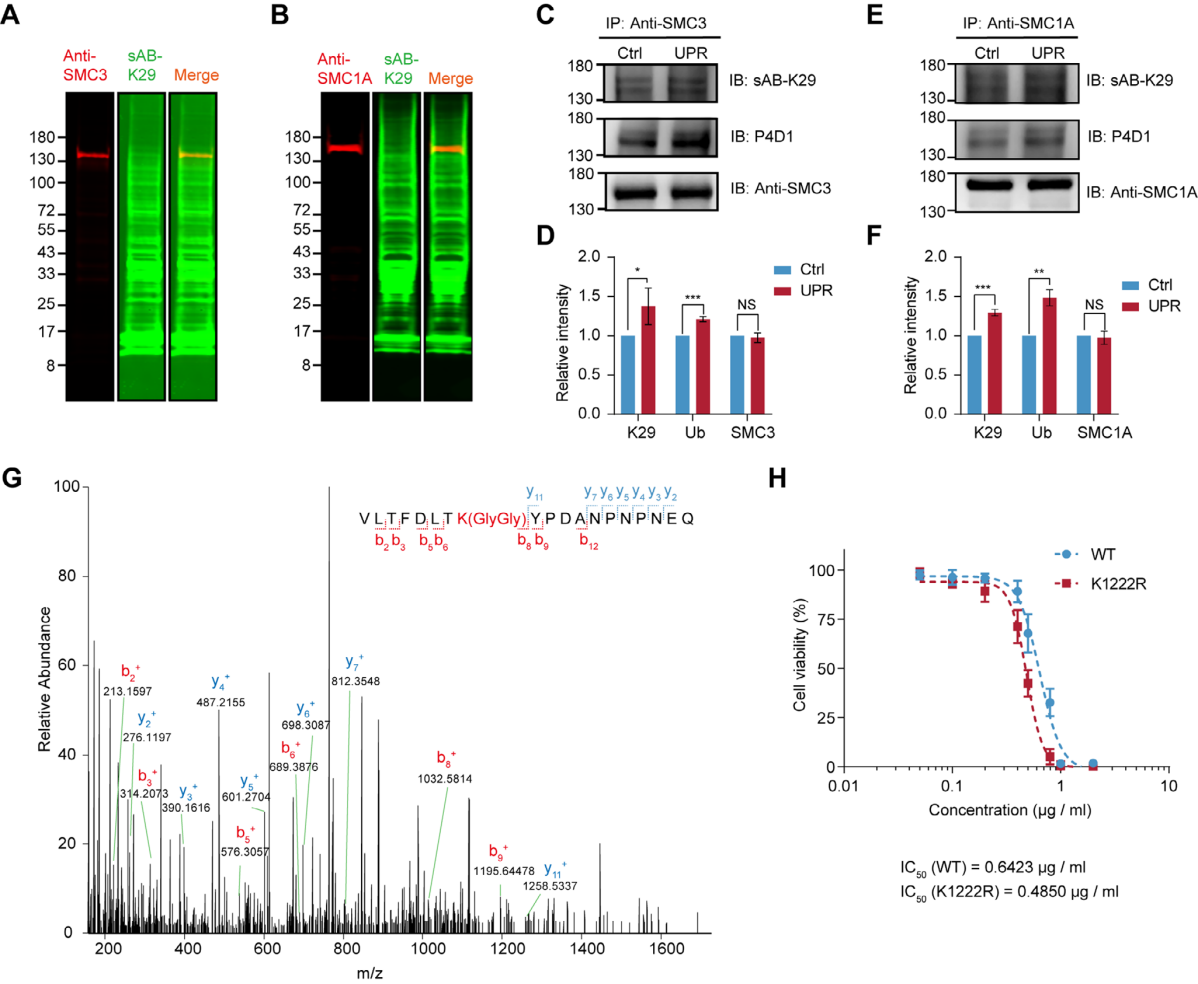
K29‐linked ubiquitination of SMC3 and SMC1A increases during the UPR. A) Fluorescence immunoblotting results of nuclear proteins with anti‐SMC3 (left panel) and sAB‐K29 (middle panel) primary antibodies. B) Fluorescence immunoblotting results for nuclear proteins with anti‐SMC1A (left panel) and anti‐sAB‐K29 (middle panel) primary antibodies. C) Immunoprecipitation (IP) using anti‐SMC3, followed by western blotting (IB) using sAB‐K29, anti‐monoubiquitin (P4D1) or anti‐SMC3 as the primary antibody. D) Relative intensity analysis of the SMC3 bands in C). (Data are presented as the means ± s.d.s, *n* = 3, two‐sided Student's *t*‐test, ns *p* > 0.05, ^*^ *p* < 0.05, ^***^ *p* < 0.001). E) Immunoprecipitation (IP) using anti‐SMC1A, followed by western blotting (IB) using sAB‐K29, anti‐monoubiquitin (P4D1) or anti‐SMC1A as the primary antibody. F) Relative intensity analysis of the SMC1A bands in E). (Data are presented as the means ± s.d.s, *n* = 3, two‐sided Student's *t*‐test, ns *p* > 0.05, ^**^ *p* < 0.01, ^***^ *p*< 0.001). G) Peptide MS/MS spectra of SMC1A [1215‐1233] under induced conditions. h) Cell viability curves of the HEK293T‐WT cell line and the HEK293T‐K1222R cell line under Tm treatment.

Therefore, to further validate which protein subunits undergo K29 ubiquitination, we conducted protein fluorescence immunoblotting experiments. The results indicated that both SMC3 and SMC1A colocalized with K29‐linked ubiquitin chains (**Figure** **4**A,B) and with monoubiquitin (Figure S7b,c, Supporting Information), confirming their K29‐linked ubiquitination. To investigate the changes in K29‐linked ubiquitination during the UPR, we extracted nuclear proteins from UPR‐induced and untreated cells and performed immunoprecipitation using anti‐SMC3 or anti‐SMC1A antibodies to enrich intracellular SMC3 or SMC1A, respectively. Through western blotting analysis of the enriched proteins, we observed a strong interaction between SMC1A and SMC3, both of which were co‐pulled down, with two bands visible when sAB‐K29 or anti‐monoubiquitin (P4D1) was used as the primary antibody (SMC1A above and SMC3 below). Furthermore, after UPR induction, total ubiquitination and K29‐linked ubiquitination of SMC1A and SMC3 tended to increase (Figure 4C–F). To further confirm the increase in K29‐linked ubiquitination and identify potential ubiquitination sites, we identified Gly‐Gly‐modified peptide segments of the enriched proteins from the above immunoprecipitation experiments. For SMC3, we did not identify a significant increase in Gly‐Gly modification in peptide segments after UPR induction. However, for SMC1A, we identified the SMC1A [1215‐1233] peptide segment with Gly‐Gly modification after UPR induction, whereas no Gly‐Gly modification was observed when the cells were not treated. Peptide MS/MS spectra of SMC1A [1215‐1233] indicated that Gly‐Gly modification was present at the K1222 site of SMC1A (Figure 4G; Figure S8, Supporting Information). The SMC1A protein consists of 1233 amino acid residues, with K1222 located at its C‐terminus within the protein‐protein and protein‐DNA interaction regions. Ubiquitination at the K1222 site likely affects the binding of the SMC1A protein to other protein subunits and DNA. Additionally, we established a HEK293T cell line with the SMC1A K1222R mutation (Figure S9, Supporting Information), which presented a significantly decreased half maximal inhibitory concentration (IC_50_) of Tm compared with that of the wild‐type HEK293T cell line, indicating the crucial role of this site in the UPR (Figure 4H).

### 2.4 K29‐Linked Ubiquitination Of Cohesin Proteins Regulates The Transcription of Cell Proliferation‐Related Genes

To investigate the transcriptional regulatory role of K29‐linked ubiquitination of cohesin during the UPR, we first utilized CUT&Tag to analyze the enrichment patterns of cohesin subunits (including SMC1A, SMC3, RAD21, STAG2, PDS5A, and PDS5B) and the cohesin release factor WAPL on chromatin.^[^ ^45^ ^]^ We focused particularly on the enrichment at the promoter regions of the downregulated gene set after UPR induction. We observed a significant decrease in the enrichment of SMC1A and RAD21 at the promoter regions of the downregulated genes, whereas the enrichment of WAPL significantly increased (Figure S10, Supporting Information). WAPL can promote the release of cohesin from chromatin.^[^ ^45^ ^]^ These results suggest that after UPR induction, the cohesin‐mediated transcription initiation complex at promoter regions of specific genes may disintegrate, leading to changes in gene transcription.

**Figure 5:**
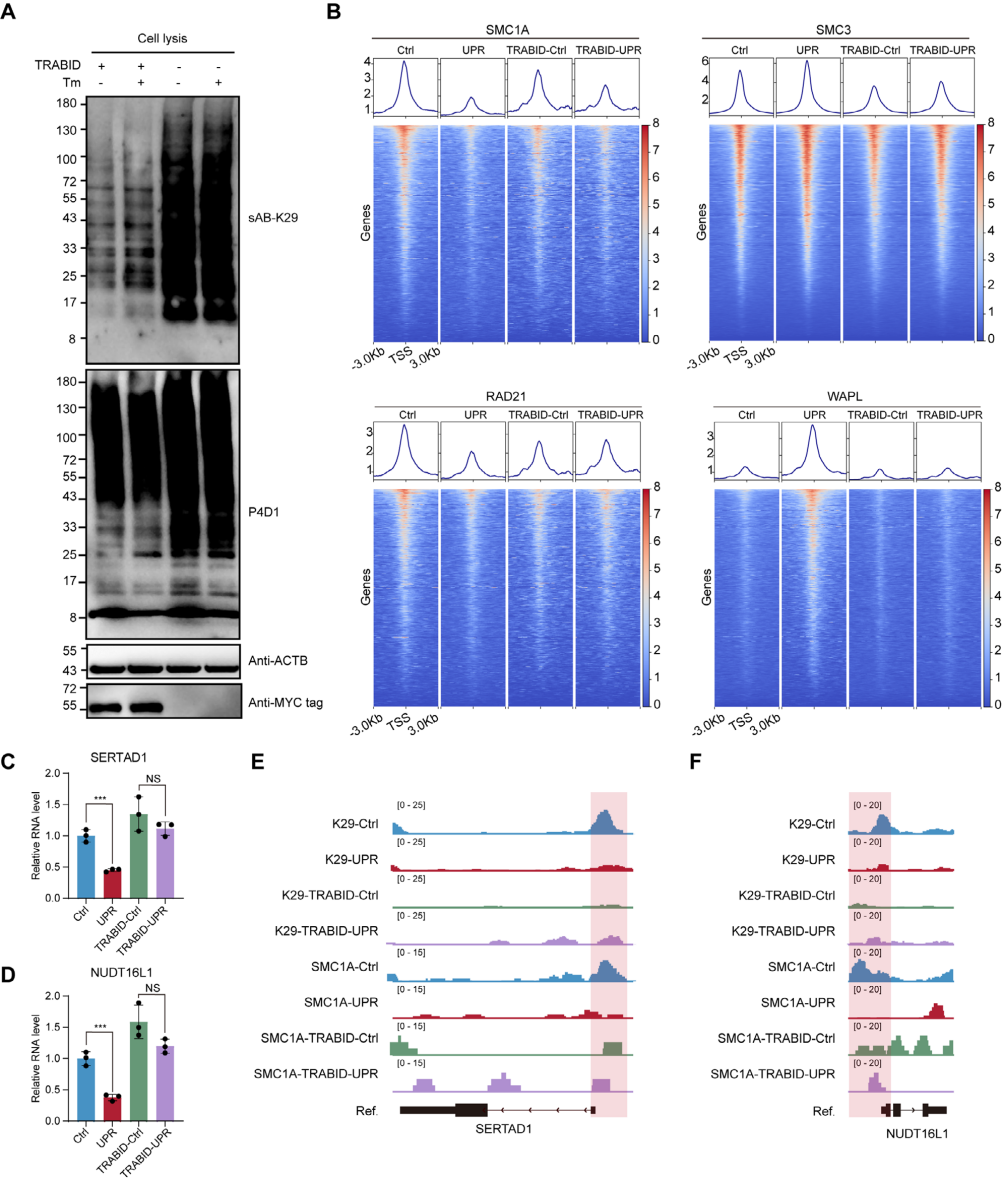
K29‐linked ubiquitination regulates the transcription of cell proliferation‐related genes. A) Total protein was extracted from TRABID‐OE‐HEK293FT cells (TRABID+) and WT‐HEK293FT cells (TRABID‐) under induced (UPR) or untreated (Ctrl) conditions, and western blotting was performed with sAB‐K29, P4D1, anti‐ACTB, or anti‐MYC tags as the primary antibodies. B) SMC1A, SMC3, RAD21, and WAPL CUT&Tag density heatmaps across all the genes of TRABID‐OE‐HEK293FT cells (TRABID) and WT‐HEK293FT cells under induced (UPR) or untreated (Ctrl) conditions. C) and D) Relative mRNA levels of SERTAD1 and NUDT16L1 in TRABID‐OE‐HEK293FT cells (TRABID) and WT‐HEK293FT cells under induced (UPR) or untreated (Ctrl) conditions. The RPKM values of SERTAD1 or NUDT16L1 were calculated from RNA‐seq data. (*n* =  3 biological replicates, mean ± s.d., two‐sided Student's *t*‐test, NS>0.05, ^***^ *p* < 0.001). E) and F) CUT&Tag peaks of K29‐linked ubiquitin chains and SMC1A around the SERTAD1 and NUDT16L1 gene loci. The red box indicates the promoter region.

To further confirm the role of K29‐linked ubiquitination, we reduced the K29‐linked ubiquitin chains in cells. To this end, we designed a plasmid containing a segment of a red fluorescent protein (mCherry) sequence and a segment of a truncated human deubiquitinase that mainly deubiquitinates K29‐linked ubiquitin chains, namely, TRABID (recombinant tumor‐necrosis factor receptor‐associated factor‐binding protein domain) with a MYC tag (Figure S11a, Supporting Information). After HEK293FT cells were transfected with this plasmid, we used flow cytometry to isolate cells expressing mCherry, indicating successful plasmid transfection. Total cellular proteins were extracted for western blotting, confirming a significant decrease in K29‐linked ubiquitin chain levels within the cells, whereas the total ubiquitin levels showed relatively minor changes (**Figure** **5**A). Immunofluorescence images also revealed a significant decrease in K29‐linked ubiquitin chain levels both inside and outside the nuclei of cells overexpressing TRABID (Figure S11b, Supporting Information). In addition, the number of K29‐linked ubiquitin chains on chromatin significantly decreased after TRABID overexpression (Figure S11c, Supporting Information). These results suggest that TRABID overexpression successfully reduced K29‐linked ubiquitin chains within the cells. Meanwhile, the K29‐linked ubiquitination of SMC1A and SMC3 was also decreased in TRABID overexpression cells (Figure S12, Supporting Information).

For the TRABID overexpression cells (TRABI‐OE‐HEK293FT), we induced the UPR and performed CUT&Tag. Compared with those in the untreated group, the decreasing trend of SMC1A and RAD21 enrichment at the transcription start sites (TSSs) in the TRABID‐OE‐HEK293FT cells was mitigated. Additionally, there was no significant enrichment of WAPL in TRABID‐OE‐HEK293FT cells after UPR induction (Figure 5B). These results indicate that K29‐linked ubiquitin chains have a significant effect on the distribution of the cohesin complex on chromatin. Moreover, these findings suggest that the K29‐linked ubiquitination of cohesin may affect its interaction with WAPL, thereby affecting the release of cohesin from chromatin. Nevertheless, the distribution of SMC3 on chromatin did not exhibit the anticipated changes (Figure 5B). We speculated that throughout this process, cohesin underwent disassembly and partially dissociated from the chromatin. Next, we investigated which specific gene transcription was affected by this mechanism.

Combining the RNA‐seq data, we observed that, in wild‐type cells, the RNA levels of the SERTAD1 and NUDT16L1 genes significantly decreased after UPR induction, whereas in TRABID‐OE‐HEK293FT cells, the induction of the UPR had no significant effect on their RNA levels (Figure 5C,D). When the TRABID‐plasmid were thoroughly removed after 14 days of culture of transfected cells, the expression of SERTAD1 and NUDT16L1 RNA returned to that observed in WT cells (Figure S13, Supporting Information). Both the SERTAD1 and NUDT16L1 genes are associated with cell proliferation, and a reduction in their expression levels can inhibit cell proliferation. After analyzing the distribution changes of K29‐linked ubiquitin chains and SMC1A at the promoter regions of SERTAD1 and NUDT16L1 (Figure 5E,F), we found that in untreated WT cells, both K29 and SMC1A were enriched at these loci, whereas in UPR‐induced WT cells, there was a significant decrease. However, in TRABID‐OE‐HEK293FT cells, the enrichment of SMC1A at these sites did not significantly diminish after UPR induction. These findings suggest that the K29‐linked ubiquitination of SMC1A may be a key regulator of the transcription levels of SERTAD1 and NUDT16L1. These results suggest that during the UPR, increased K29‐linked ubiquitination of the cohesin complex leads to the transcriptional downregulation of genes related to cell proliferation and ultimately the inhibition of cell proliferation.

## 3 Conclusion

The unfolded protein response is a crucial mechanism for the survival of cells under endoplasmic reticulum stress and involves intricate regulation of gene transcription, RNA translation, protein folding, and degradation. During the UPR process, cell growth halts, redirecting energy to repair the abnormal state of the endoplasmic reticulum, but the regulatory mechanisms of relevant transcriptional levels remain unclear. Gene transcription is regulated by transcription regulators and corresponding enzymes. Ubiquitination, an important protein modification, is highly likely to modify relevant transcription regulators to regulate downstream transcription. Therefore, we investigated the relationship between the transcriptional downregulation of cell growth‐related genes and their ubiquitination during the UPR.

In this study, through CUT&Tag and ATAC‐seq, we observed significant enrichment of K29‐linked ubiquitin chains at accessible chromatin loci and promoter regions, indicating a close association of K29‐linked ubiquitination with transcriptional regulation. Immunofluorescence and CUT&Tag revealed a marked decrease in the number of K29‐linked ubiquitin chains in the nucleus after UPR induction, particularly in the promoter regions of genes whose transcription levels were downregulated. These findings suggest a unique regulatory role of K29‐linked ubiquitin chains in the transcriptional downregulation of genes during the UPR. As protein modifications, K29‐linked ubiquitin chains likely exert their regulatory influence on transcription by modifying transcription regulators. To identify potential transcription factors regulated by K29‐linked ubiquitin chains during the UPR, we predicted possible factors and performed mass spectrometry analysis of nuclear proteins enriched with biotinylated TUBEs and sAB‐K29. We found that the cohesin complex, as a potential transcriptional regulator, significantly increased K29‐linked ubiquitination after UPR induction.

Because there is a marked decrease in the number of K29‐linked ubiquitin chains in the nucleus after UPR induction, we did indeed focus more on K29‐downregulated proteins. However, based on our predictions of potential transcription factors involved in UPR (Table S1, Supporting Information), none of the K29‐downregulated proteins were potential transcription factors. Instead, we identified potential transcription factors among the K29‐upregulated proteins. Due to the relatively low levels of transcription factors, their contribution to the overall K29 level is minimal, and the observed increase in their K29‐linked ubiquitination does not conflict with the overall decrease in K29‐linked ubiquitination.

Through immunoblotting experiments, we confirmed K29‐linked ubiquitination of the cohesion subunits SMC1A and SMC3, with the potential ubiquitination site K1222 identified on SMC1A through mass spectrometry. To further elucidate the regulatory mechanism of K29‐linked ubiquitin chains, we generated K29‐depletion cells. By integrating the chromatin landscapes of various subunits of the cohesin complex and transcriptomic data, we identified the SERTAD1 and NUDT16L1 genes as downstream regulatory targets associated with cell proliferation and further confirmed that K29‐linked ubiquitination can regulate transcription by modifying the cohesin complex during the UPR. The ubiquitylation of cohesin may lead to its mobilization from chromatin, thus facilitating stalled replication fork dynamics.^[^ ^46^ ^]^ These findings indicate that ubiquitination of cohesin can alter the interactions between cohesin and other molecules. Overall, we propose a potential regulatory mechanism: during the UPR, the increase in K29‐linked ubiquitination on the cohesin complex leads to the release of cohesin and disintegration of the transcription initiation complex at gene promoter regions, resulting in the transcriptional downregulation of genes involved in cell proliferation and the consequent inhibition of cell growth.

The K29‐linked ubiquitination‐mediated regulation of cohesin dynamics provides insights into the intricate mechanisms by which cells modulate gene expression in response to environmental cues, such as the UPR. In this study, we elucidated a novel transcriptional regulatory mechanism associated with cell growth arrest during the UPR process, identifying potential therapeutic targets for UPR‐related diseases. Unlike its traditional role as a degradation signal, ubiquitin plays a new role in the UPR. The discovery of a novel transcriptional regulatory function of the non‐canonical K29‐linked ubiquitin chain has broadened our understanding of non‐canonical ubiquitin chains. Furthermore, the cohesin complex, a crucial protein that regulates chromatin structure, plays significant roles in sister chromatid separation and chromatin organization. Here, we propose that K29‐linked ubiquitination of cohesin may impact its mobility on chromatin, thereby regulating the transcriptional levels of associated genes. This modification could also potentially influence the execution of other functions by the cohesin complex, offering a new perspective on the chromatin structural regulatory mechanism of cohesin and the pathogenesis of cohesin‐related diseases.

If adaptive UPR responses fail to restore protein‐folding homeostasis, the UPR signal will continue to exist and eventually transform into the “terminal UPR,” which ultimately promotes apoptosis.^[^ ^35^ ^]^ Whether ubiquitination or cohesin plays a crucial role in this process remains to be studied. In addition, other cellular stress responses, such as the heat shock protein response (HSP) and the hypoxic stress response, may also involve the transcriptional regulatory mechanism reported in this work. In addition, how K29‐linked ubiquitination affects the structure of the cohesin complex at the microscopic level and how its upstream regulatory signaling pathway is activated remain to be studied.

## 4 Experimental Section

### Cell Culture

The HEK293FT cell line was obtained from the Cell Resource Center, Institute of Basic Medical Sciences, CAMS/PUMC (Catalog# 1101HUM‐PUMC000364, RRID: CVCL_6911). The cell lines were checked free of mycoplasma contamination by PCR. Their species origins were confirmed with PCR. The identity of the cell line was authenticated with STR profiling (FBI, CODIS). All the results can be viewed on the website (http://www.cellresource.cn). HEK293FT cells were cultured in Dulbecco's modified Eagle's medium (DMEM, Gibco, 11995065) supplemented with 10% fetal bovine serum (FBS, Yeasen, 40130ES76), 1 × penicillin‐streptomycin (Yeasen, 60162ES76), 1 × MEM non‐essential amino acids solution (Gibco,11140035), and 1× GlutaMAX (Gibco, 35050061) at 37 °C with 5% CO_2_.

### Plasmid Construction

Lentivirus packaging plasmid pMD2.G (Addgene #12259) and psPAX2 (Addgene #12260) were gifts from Didier Trono. TUBEs expression plasmid and pTRABID‐mCherry plasmid were synthesized by Anhui General Biology, China.

### Cell Transfection

HEK293FT cells were cultured in 90 mm Petri dishes for 70–90% confluence. The medium was replaced with 6 mL of antibiotic‐free DMEM (DMEM with 10% FBS, 1 × MEM and 1 × GlutaMAX) 30 min before transfection. A total of 40 µL of Lipofectamine 3000 transfection reagent (Invitrogen, L3000015) was diluted in 1.5 mL of Opti‐MEM I (Gibco, 31985070) and incubated at room temperature for 5 min. Then, 15 µg of plasmid was diluted in 1.5 mL of Opti‐MEM, mixed with the diluted Lipofectamine 3000 transfection reagent, and incubated at room temperature for 20 min. After incubation, 3 mL of the mixture was added to the medium, and the cells were incubated at 37 °C with 5% CO_2_.

### Flow Cytometry Cell Sorting

HEK293FT cells were transfected with the pTra‐mCherry plasmid. After 24 h of transfection, the medium was replaced with DMEM containing 2 µg mL^−1^ tunicamycin or an equal amount of DMSO as a control. After 24 h of incubation, the cells were digested with 0.05% trypsin (0.25% trypsin (Gibco, 15050065) diluted in 1 × PBS) at 37 °C for 3 min. Then, the cells were collected by centrifugation and resuspended in DMEM. Then, the cells were sorted with a Beckman Moflo AstriosEQ, and the cells expressing mCherry were collected for CUT&Tag and ATAC analysis.

### Protein Expression and Purification

The sAB‐K29 antibody, biotinylated sAB‐K29, and biotinylated TUBEs were expressed following established protocols.^[^ ^13^ ^]^ Briefly, the plasmids encoding sAB‐K29, sAB‐K29‐LPETGG‐His, or TUBEs‐LPETGG‐His were transformed into T7pLysY competent cells (Biomed, BC207) and induced with 0.25 mm isopropyl β‐d‐1‐thiogalactopyranoside (IPTG, Yeasen, 10902ES08) at 16 °C for 20 h. The cells were harvested, resuspended, sonicated, and centrifuged. sAB‐K29 was purified on a protein G‐Sepharose column (Cytiva). sAB‐K29‐LPETGG‐His or TUBEs‐LPETGG‐His was captured by the chitin resin and eluted with 100 mm DTT. The proteins were dialyzed overnight at 4 °C using dialysis buffer (100 mm HEPES‐KOH pH 7.5, 200 mm NaCl, 0.2 mm EDTA, 2 mm DTT, 0.2% Triton X‐100, 20 µm PMSF, and 20% glycerol). Then, sAB‐K29‐LPETGG or TUBEs‐LPETGG was mixed with 150 µm GGGGGK‐Biotin, 10 µm Sortase A (MCE, HY‐E70234) and 5 mm CaCl_2_ and incubated at room temperature for 1 h. After incubation, the proteins were dialyzed overnight at 4 °C using dialysis buffer to remove GGGGGK‐Biotin.

### Extraction of Nuclear Protein

The cells were cultured in 150 mm Petri dishes and washed three times with 10 mL of pre‐cooled 1 × PBS. The cells were subsequently collected with a cell spatula. Nuclear protein was extracted via the Nuclear and Cytoplasmic Protein Extraction Kit (Yeasen, 20126ES60). For each 150 mm dish, 3 mL of Cytoplasmic Extraction Reagent A, 150 µL of Cytoplasmic Extraction Reagent B, and 750 µL of Nuclear Extraction Reagent were used. Before extraction, 10 µm PR‐619 (MCE, HY‐13814), 10 µm 1,10‐phenanthroline (MCE, HY‐W004544), and 1 mm PMSF were added to Cytoplasmic Extraction Reagent A and Nuclear Extraction Reagent. After the addition of Nuclear Extraction Reagent, the sample was sonicated at 4 °C for 16 min (3 s on, 9 s off, 80% power). The sample was subsequently centrifuged for 10 min (16 000 × g, 4 °C), and the supernatant was collected and stored at −80 °C after the protein concentration was determined via a BCA protein quantification kit (Yeasen, 20200ES76).

### Immunoprecipitation for Mass Spectrometry

200 µL of streptavidin‐magnetic beads were washed three times with 1 × PBS and then incubated with 60 µg of Biotin‐TUBEs on a rotator at room temperature for 1 h. Then, the beads were washed with 500 µL of 1× PBS three times and incubated with 1 mg of cell nuclear protein on a rotator at 4 °C overnight. Next, the beads were washed with 500 µL of PBST (1× PBS with 0.05% Tween 20) for 5 min on a rotator at 4 °C four times. Then, 100 µL of 50 mm NaOH was added, and the mixture was eluted for 10 min at room temperature. The eluate was collected into a new tube and neutralized with 50 µL of 1 m Tris‐HCl (pH 7.5). Another 200 µL of streptavidin‐magnetic beads was washed three times with 1× PBS and then incubated with 60 µg of biotin‐K29 antibody on a rotator at room temperature for 1 h. After being washed with 1× PBS, the beads were incubated with 150 µL of the neutralizing solution at 4 °C on a rotator overnight. Next, the beads were washed with 500 µL of PBST (1× PBS with 0.05% Tween) for 5 min on a rotator at 4 °C four times. Then, 20 µL of 50 mm NaOH was added, and the mixture was eluted for 10 min at room temperature. The eluate was collected into a new tube and neutralized with 10 µL of 1 m Tris‐HCl (pH 7.5). Then, the neutralized solution was mixed with 7.5 µL of 5 × SDS‒PAGE protein loading buffer and boiled at 100 °C for 10 min. The samples were separated via SDS‐PAGE.

### Mass Spectrometry

The gel bands of interest were excised from the gel, which was followed by in‐gel digestion with sequencing‐grade modified trypsin at 37 °C overnight. The peptides were extracted twice with 0.1% trifluoroacetic acid in 50% acetonitrile aqueous solution for 30 min and then dried in a speedvac. Peptides were re‐dissolved in 25 µL of 0.1% trifluoroacetic acid, and 6 µL of extracted peptides were analyzed via Thermo Orbitrap Fusion.

For LC‐MS/MS analysis, the peptides were separated via 60 min gradient elution at a flow rate of 0.30 µL min^−1^ with an EASY‐nLC 1000 system, which was directly interfaced with an Orbitrap LUMOS mass spectrometer (Thermo Fisher Scientific, Bremen, Germany). The analytical column was a custom‐made fused silica capillary column (75 µm ID, 150 mm length; Upchurch, Oak Harbor, WA) packed with C‐18 resin (300 Å, 5 µm, Varian, Lexington, MA). The mobile phase consisted of 0.1% formic acid, and mobile phase B consisted of 100% acetonitrile and 0.1% formic acid. The Orbitrap Fusion mass spectrometer was operated in the data‐dependent acquisition mode via Xcalibur 3.0 software, and there was a single full‐scan mass spectrum in the Orbitrap (350‐1550 m/z, 120 000 resolution) followed by top‐speed MS/MS scans in the Orbitrap.

The MS/MS spectra from each LC‐MS/MS run were searched against the target protein database from UniProt via an in‐house Proteome Discoverer (version PD3.0; Thermo Fisher Scientific, USA). The search criteria were as follows: full chymotrypsin specificity was needed; four missed cleavages were allowed; oxidation (M) and 54.01063 Da F) were set as the variable modifications; precursor ion mass tolerances were set at 20 ppm for all MS acquired in an Orbitrap mass analyzer; and the fragment ion mass tolerance was set at 0.02 Da for all MS2 spectra acquired. The peptide false discovery rate (FDR) was calculated via the fixed value PSM validator provided by the PD. When the q value was smaller than 1%, the peptide spectrum match (PSM) was considered to be correct. FDRs were determined on the basis of PSMs when searching against the reverse decoy database. Peptides assigned to only a given protein group were considered unique. The false discovery rate (FDR) was also set to 0.01 for protein identification.

### Immunoprecipitation for Western Blotting

30 µL of rProtein A/G MagBeads (Yeasen, 36417ES03) were washed three times with 1 × PBS and then incubated with 5 µL of anit‐SMC1 (Invitrogen, 38597A12) or anti‐SMC3 (Abcam, AB9263) antibody diluted with 45 mL 1× PBS on a rotator at room temperature for 0.5 h. Then, the beads were washed with 200 µL of 1 × PBS three times and incubated with 150 µg of cell nuclear protein on a rotator at RT for 1 h. Next, the beads were washed with 200 µL of PBST (1 × PBS with 0.05% Tween 20) for three times. Then, 75 µL of 1 × SDS‐PAGE protein loading buffer was added and boiled at 100 °C for 10 min. The samples were separated by SDS‐PAGE in three lanes (25 µL in each lane), and transferred to the nitrocellulose membrane for western blotting using sAB‐K29, P4D1, anti‐SMC1 / SMC3 as primary antibodies.

### Western Blotting

Proteins were mixed with 5 ×SDS‐PAGE protein loading buffer, boiled for 10 min, and separated via SDS‒PAGE. After being transferred to a nitrocellulose membrane, the membrane was washed with 1 × TBST for 5 min. Then, it was blocked with 5% skim milk at room temperature for 1 h. The membrane was subsequently washed with 1 × TBST for 5 min three times and incubated in primary antibody solution at room temperature for 1 h. After incubation, it was washed with 1 × TBST buffer 3 times and incubated in secondary antibody solution at room temperature for 1 h. Finally, after three washes with PBST, the protein in the membrane was detected with a Super ECL Detection Reagent ECL Kit (Yeasen, 36208ES76) and imaged with a BLT GelView 6000 Plus.

When the primary antibody was sAB‐K29 (1:1500), the secondary antibody was rabbit anti‐human IgG F(ab')2/HRP (1:2000, Bioss, bs‐0364R‐HRP). The primary antibodies used were anti‐monoubiquitin (P4D1) (1:200, Santa Cruz, sc‐8017), anti‐PDS5A (1:2000, Invitrogen, YB3837049), anti‐WAPL (1:200, Santa Cruz, sc‐365189), anti‐STAG2 (1:200, Santa Cruz, sc‐81852), anti‐RAD21 (1:200, Santa Cruz, sc‐166973), anti‐MYC tag (1:2000) and anti‐PDS5B (1:200, Santa Cruz, sc‐81635), the secondary antibody used was goat anti‐mouse IgG H&L/HRP (1:2000, Bioss, bs‐40296G‐HRP). When the primary antibodies used were anti‐SMC3 (1:2000, Abcam, AB9263), anti‐SMC1 (1:2000, Invitrogen, 38597A12), anti‐M1 linkage (1:1000, Novus Biologicals, NBP3‐05631), anti‐K33 linkage (1:1000, Novus Biologicals, NBP3‐05656), anti‐K6 linkage (1:1000, Novus Biologicals, NBP3‐05680), anti‐K11 linkage (1:2000, Sigma–Aldrich, SAB5701121), anti‐K48 linkage (1:2000, Abcam, ab140601), anti‐K63 linkage (1:2000, Abcam, ab179434) and anti‐K27 linkage (1:2000, Abcam, ab181537), the secondary antibody used was goat anti‐rabbit IgG H&L/HRP (1:2000, Bioss, bs‐0295G‐HRP).

### Fluorescent Western Blotting

To demonstrate the ubiquitination of SMC1A/SMC3, the sample was transferred to a nitrocellulose membrane and blocked with 5% skim milk. Then, the membrane was washed with 1 × TBST for 5 min three times and incubated with primary antibody mixture (anti‐monoubiquitin (P4D1) antibody (1:200) and anti‐SMC1A/anti‐SMC3 (1:2000)) at room temperature for 1 h. Next, the membrane was washed with 1 × TBST buffer 3 times, incubated with fluorescent antibody mixture (anti‐mouse‐700 (1:10000) and anti‐rabbit‐800 (1:10000)) for 1 h at room temperature, and imaged with an Odyssey DLX imaging system.

To demonstrate the K29‐linked ubiquitination of SMC1A/SMC3, the sample was transferred to a nitrocellulose membrane and blocked with 5% skim milk. Then, the membrane was washed with 1 × TBST for 5 min three times and incubated with primary antibody mixture (sAB‐K29 antibody for 1:1500, anti‐SMC1A/anti‐SMC3 for 1:2000) at room temperature for 1 h. Next, the membrane was washed with 1× TBST buffer 3 times, incubated with secondary antibody (goat anti‐human IgG F(ab')2 for 1:2000) at room temperature for 1 h. Finally, the membrane was incubated with fluorescent antibody mixture (anti‐goat‐700 (1:10000), anti‐rabbit‐800 (1:10000)) for 1 h at room temperature and imaged with an Odyssey DLX imaging system.

### Immunofluorescence Imaging

HEK293FT cells at an appropriate density were passed through a 96‐well glass‐bottom plate. The cells were treated with 4% (w/v) paraformaldehyde (Yeasen, 60536ES60) for 20 min at room temperature for fixation and washed once with 1× PBS. The fixed cells were permeabilized and blocked with 0.5% Triton X‐100 (Beijing Dingguo Changsheng Biotechnology, AR‐0341) and 1% BSA in PBS for 20 min at room temperature. Then, the cells were washed once with PBS and incubated in primary antibody solution (1:100) at room temperature for 1 h. After three washes with PBS, they were incubated in secondary antibody solution (1:100) at room temperature for 1 h. Next, the cells were washed with PBS three times, mounted with DAPI Fluoromount‐G (SouthernBiotech, 0100–20), and imaged.

When sAB‐K29 was used as the primary antibody, the secondary antibody was FITC‐conjugated rabbit anti‐human IgG F(ab')2 (Bioss, bs‐0364R‐FITC). When the primary antibodies were anti‐K27, anti‐K48, or anti‐K63, the secondary antibody was FITC‐conjugated goat anti‐rabbit IgG H&L (Bioss, bs‐0295G‐FITC). When the primary antibody was anti‐monoubiquitin (P4D1), FITC‐conjugated rabbit anti‐mouse IgG H&L (Bioss, bs‐0296R‐FITC) was used as the secondary antibody.

All images were acquired via a Lecia STELLARIS 5 confocal microscope (Leica, Germany) with a 63 × oil‐immersion objective. The image size was 184.52 µm × 184.52 µm, and the single pixel size was 180 nm × 180 nm. DAPI was excited with UV (405 nm) and detected with a 430–500 nm bandpass filter. FITC was excited with a white laser (488 nm) and detected with a 500–535 nm bandpass filter.

### Construction of SMC1A K1222R Cell Line

SMC1A K1222R cell line was constructed using CRISPR gene knock‐in technology. Briefly, the plasmid and oligo were transferred into cells by electroporation. The sequence of the gRNA and the donor oligo are listed in Table S3 (Supporting Information). The generation of monoclonal was achieved through limiting dilution in 96‐well plates. After incubation, DNA extraction was carried out, followed by PCR and sanger sequencing. The correctly identified cells were further cultured and expanded.

### RNA Extraction and RT‐qPCR

Total RNA was extracted from the cells using the RNA Easy Fast Tissue/Cell Kit (TIANGEN; DP451) according to the manufacturer's protocol. The reverse transcription was performed using random primers and the Hifair II 1st Strand cDNA Synthesis Kit (Yeasen, 11119ES60) according to the manufacturer's protocol. qPCR was conducted via Hieff qPCR SYBR Green Master Mix (Yeasen, 11201ES03) following the manufacturer's protocol. The primers used for qPCR are listed in Table S4 (Supporting Information). Ct values were measured via a Bio‐Rad CFX96 (Bio‐Rad) instrument and were averaged from 3 replicate measurements. The housekeeping gene Gapdh was used as an internal control, and the expression levels of the target genes were calculated via the 2^–ΔΔCt^ method.

### RNA‐seq

The cells were washed twice with pre‐cooled 1 × PBS. Total RNA was subsequently extracted with TRIzol (BIORIGIN, BN20537). An RNA‐seq library was constructed via the NEBNext Ultra II RNA Library Prep Kit for Illumina (NEB, E7770S) according to the manufacturer's protocol. Bulk RNA‐seq libraries were sequenced via the 150 bp paired‐end Illumina NovaSeq 6000 platform.

### RNA‐seq Data Analysis

RNA‐seq data analysis was performed by NovelBio Co., Ltd. with CytoNavigator SingleCell Analysis Platform (sc.novelbrain.com).

### CUT&Tag

CUT&Tag was performed following the established protocol^[^ ^36^ ^]^ with some modifications. Briefly, ≈50 000 cells were collected and resuspended in 90 µL of 1 × Wash Buffer A (20 mm HEPES‐KOH pH 7.5, 150 mm NaCl, 0.5 mm spermidine, and 1 × protease inhibitor). Moreover, 10 µL of Concanavalin A‐coated magnetic beads (Novoprotein, N251‐01A) were resuspended in 10 µL of 1 × binding buffer (20 mm HEPES‐KOH pH 7.5, 10 mm KCl, 1 mm MnCl_2_, and 1 mm CaCl_2_). The beads were then added to the cell suspension, mixed gently, and incubated at room temperature for 10 min while rotating. The supernatant was removed on a magnetic separator, and the beads were resuspended in 50 µL of primary antibody buffer (0.05% digitonin, 0.1% BSA, 2 mm EDTA, and 10 µg mL^−1^ primary antibody) and incubated at room temperature for 1 h while rotating. Next, the primary antibody buffer was removed, and 100 µL of secondary antibody buffer (0.05% digitonin, 0.1% BSA, 2 mm EDTA, and 10 µg mL^−1^ secondary antibody) was added. After 1 h of incubation at room temperature while rotating, the beads were washed twice with 500 µL of 1 × Wash Buffer A containing 0.05% digitalis saponin for 5 min each. Then, 100 µL of Tn5 buffer (20 mm HEPES‐KOH pH 7.5, 300 mm NaCl, 0.5 mm spermidine, 1 × protease inhibitor, 0.01% digitonin, 0.2 µm pAG‐transposome (Novoprotein, M059‐YH01‐01A)) was added, and the beads were incubated for 1 h at room temperature while rotating. The beads were washed twice with 500 µL of Wash Buffer B (20 mm HEPES‐KOH pH 7.5, 300 mm NaCl, 0.5 mm spermidine, 1 × protease inhibitor, 0.01% digitalis saponin) for 5 min each, followed by the addition of Wash Buffer B containing 10 mm MgCl_2_. After incubation at 37 °C for 1 h, 1 µL of 10% SDS was added, mixed, and incubated at 55 °C and 800 rpm on a shaker for 10 min. Then, the supernatant was purified with VAHTS DNA Clean Beads (Vazyme, N411‐03). PCR amplification was performed with 5 × Hieff Canace PCR Master Mix (Yeasen, 10137ES03) and a TruePrep Index Kit V2 (Vazyme, TD202). Finally, the library was purified via VAHTS DNA Clean Beads for high‐throughput sequencing (Illumina, NovaSeq X).

### ATAC‐seq

ATAC‐seq was performed following an established protocol with some modifications.^[^ ^37^ ^]^ Briefly, the cells were digested with 0.25% trypsin‐EDTA, washed twice with 1 × PBS, and resuspended in NEI buffer (20 mm HEPES‐KOH pH 7.9, 10 mm KCl, 2 mm MgCl_2_, 0.05 mm spermidine, 0.1% Triton X‐100, 20% glycerol) on ice for 10 min. After centrifugation at 1300 × g for 4 min at 4 °C, the supernatant was removed, and the cells were washed with PBS‐Mg buffer (1 × PBS containing 2 mm MgCl_2_) and centrifuged at 1300× g for 4 min at 4 °C. The precipitate was subsequently resuspended in PBS‐Mg buffer and counted via a Cellometer Mini (Nexcelom Bioscience). A total of 1× 10^4^ cells was collected and gently mixed with Tn5 reaction buffer (1x Reaction Buffer, 0.6 µm Tn5 transposase (Novoprotein, M045‐01B)) and incubated at 37 °C for 45 min with shaking every 10 min. The reaction was then terminated by adding 1 µL of 10% SDS and incubating at 55 °C for 10 min. Next, the DNA fragments were purified with VAHTS DNA Clean Beads and subjected to PCR amplification with 5× Hieff Canace PCR Master Mix and a TruePrep Index Kit V2. Finally, the library was purified via VAHTS DNA Clean Beads for high‐throughput sequencing.

### CUT&Tag and ATAC‐seq Data Analysis

Original fastq data were trimmed using Trim Galore software (https://www.bioinformatics.babraham.ac.uk/projects/trim_galore/, version: 0.4.4) to remove adapters and low‐quality reads (parameters: –trim1 ‐paired). Trimmed reads were filtered for repeat elements using sequences obtained from RepBase (v26.08) with Bowtie2 (v.2.3.5.1).^[^ ^47^ ^]^ The filtered reads were aligned with Bowtie2 (v.2.3.5.1) using ‐I 25 ‐X 500 ‐N 1 ‐L 25 and Samtools (v1.10).^[^ ^48^ ^]^ BAM files were deduplicated with Picard (https://broadinstitute.github.io/picard/, v2.23.4) MarkDuplicates. Normalized coverage tracks were generated using deepTools (v3.3.2)^[^ ^49^ ^]^ bamCoverage using parameters ‐binSize 10 –normalizeUsing RPKM and the respective genome size. The genome used in this study were hg19. Peaks were called with MACS2 (v2.2.6)^[^ ^50^ ^]^ (parameters: callpeak ‐f BAMPE –nomodel –nolambda –q 0.01). High confidence peaks sets were concordant peaks between two or three replicates generated with Bedtools (v2.29.2)^[^ ^51^ ^]^ intersect command. Peak overlap was plotted with Intervene^[^ ^52^ ^]^ software venn command. Peak annotation and genome distribution were generated by ChIPseeker,^[^ ^53^ ^]^ a R package. Heatmaps were generated with deepTools (v3.3.2) plotCorrelation. IGV^[^ ^54^ ^]^ was used for peak visualization.

### Statistical Analysis

For the statistical analysis of Western blotting results, ImageJ was used to calculate the gray scale of the target bands. For each repetition, the relative intensity was calculated as:

(1) $$\begin{array}{ccc} \mathrm{Rela}\mathrm{tive}\;\mathrm{inte}\mathrm{nsity} & = & \frac{\mathrm{gray}\;\mathrm{scale}\;\left(\mathrm{ubiq}\mathrm{uiti}\mathrm{nati}\mathrm{on}\;\mathrm{of}\;\mathrm{expe}\mathrm{rime}\mathrm{ntal}\;\mathrm{group}\right)}{\mathrm{gray}\;\mathrm{scale}\;\left(\mathrm{input}\;\mathrm{of}\;\mathrm{expe}\mathrm{rime}\mathrm{nt}\;\mathrm{group}\right)}\\ & & ÷\frac{\mathrm{gray}\;\mathrm{sacle}\;\left(\;\mathrm{ubiq}\mathrm{uiti}\mathrm{nati}\mathrm{on}\;\mathrm{of}\;\;\mathrm{cont}\mathrm{rol}\;\mathrm{gourp}\right)}{\mathrm{gray}\;\mathrm{sacle}\;\left(\mathrm{input}\;\mathrm{of}\;\mathrm{cont}\mathrm{rol}\;\mathrm{gourp}\right)} \end{array}$$

For the statistical analysis of RT‐qPCR results, the relative RNA expression levels were calculated as:

(2) $$\begin{array}{ccc} \Delta Ct_{1} & = & Ct\left(target\;gene\;of\;experimental\;group\right)\\ & & -Ct\left(GAPDH\;of\;experimental\;group\right) \end{array}$$

(3) $$\Delta Ct_{0}=Ct\left(target\;gene\;of\;control\;group\right)-Ct\left(GAPDH\;of\;control\;group\right)$$

(4) $$Relative\;RNA\;expression=\frac{2^{-\Delta Ct_{1}}}{Mean\left(2^{-\Delta Ct_{0}}\right)}$$

Data are presented as the mean ± SD., *n* = 3, two‐sided Student's *t*‐test using Microsoft Office 2021(Excel), ns *p* > 0.05, ^*^ *p* < 0.05, ^**^ *p* < 0.01, ^***^ *p* < 0.001, ^****^ *p* < 0.0001.

## Conflict of Interest

The authors declare no conflict of interest.

## Supporting information



Supporting Information

## Data Availability

The data that support the findings of this study are available from the corresponding author upon reasonable request.
