# Supplementary material for: K29‐Linked Ubiquitination of Transcription Regulators Controls Cell Proliferation in the Unfolded Protein Response
Source: Adv Sci (Weinh). 2025 Aug 30;12(42):e09817. doi: 10.1002/advs.202509817 (PMC12622412; doi:10.1002/advs.202509817)
Supplement: Supplementary file 1 — Supporting Information [file ADVS-12-e09817-s001.docx]

Supporting Information

**K29-linked ubiquitination of transcription regulators controls cell proliferation in the unfolded protein response**

Qiushuang Zhang, Xucong Teng*, Yicong Dai, Yuncong Wu, Hongwei Hou*, Jinghong Li*


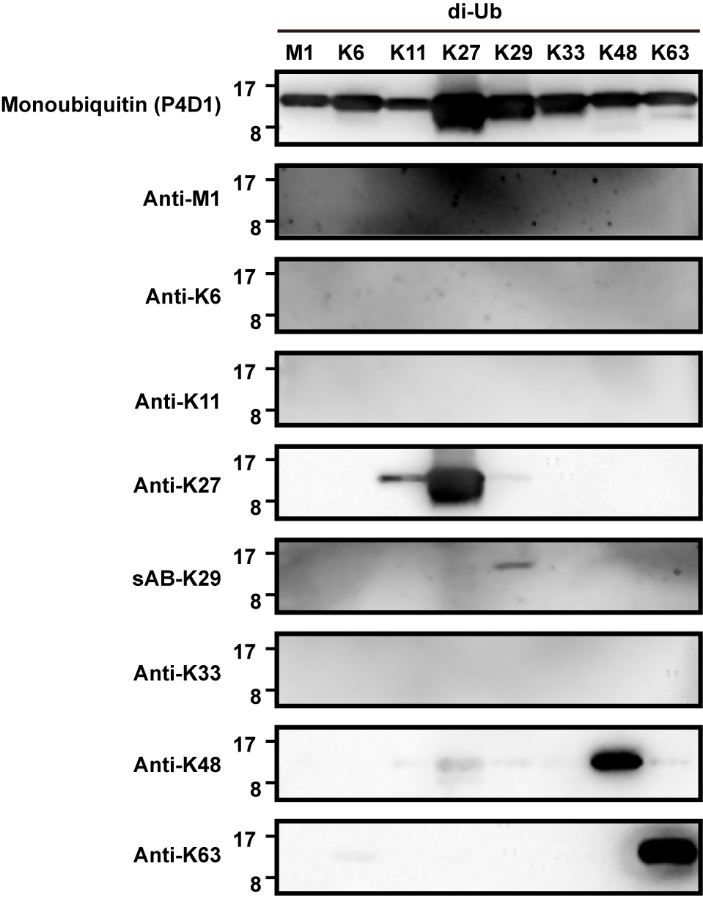


**Figure S1.** Verification of antibody specificity for each ubiquitin chain. M1-Linked di-ubiquitin (LifeSensors, LSS-SI-0102-0100), K6-linked di-ubiquitin (LifeSensors, LSS-SI-0602-0025), K11-linked di-ubiquitin (LifeSensors, LSS-SI-1102-0050), K27-linked di-ubiquitin (LifeSensors, LSS-SI-2702-0025), K29-linked di-ubiquitin (LifeSensors, LSS-SI-2902-0025), K33-linked di-ubiquitin (LifeSensors, LSS-SI-3302-0025), K48-linked di-ubiquitin (LifeSensors, LSS-SI-4802-0100), K63-linked di-ubiquitin (LifeSensors, LSS-SI-6302-0050).


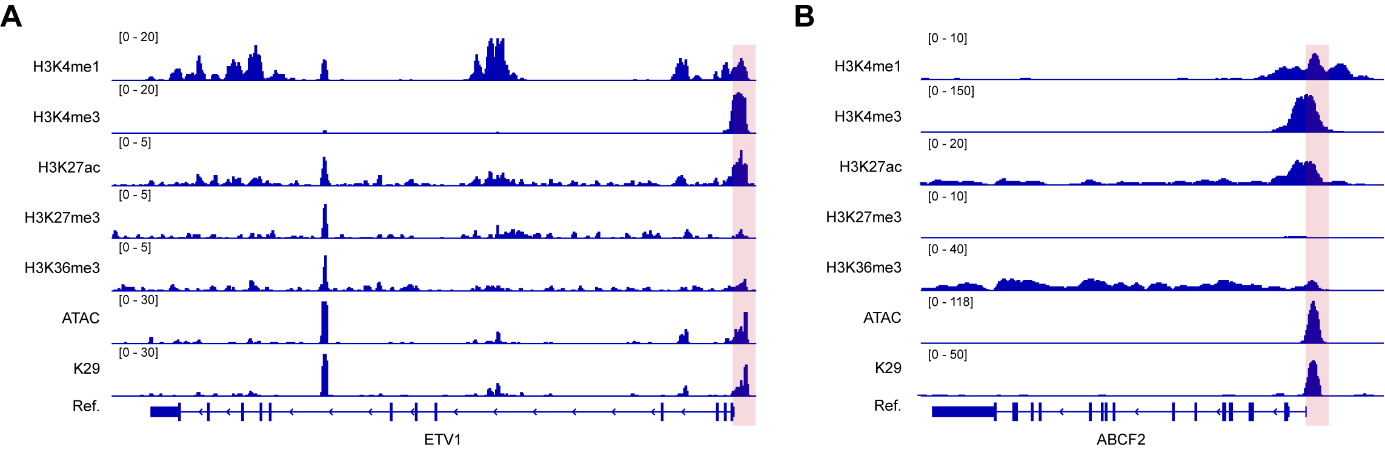
Figure S2. ATAC peaks and CUT&Tag peaks for the five histone modifications and the K29-linked ubiquitin chain around ETV1 and ABCF2 genes. The red box indicates the promoter region.


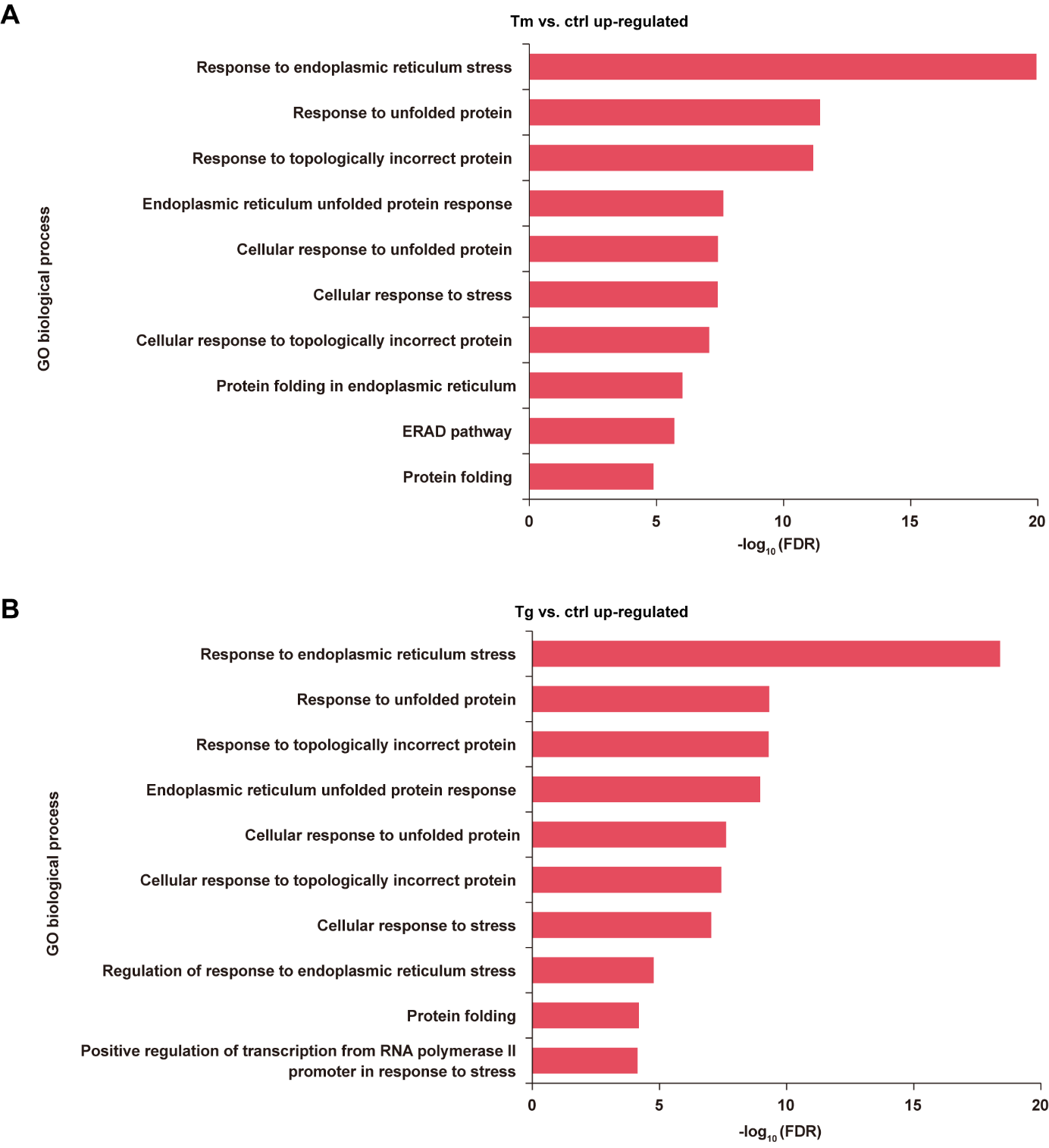
**Figure S3.** Gene ontology analysis of upregulated mRNAs from HEK293FT cells treated with A) tunicamycin or B) thapsigargin.


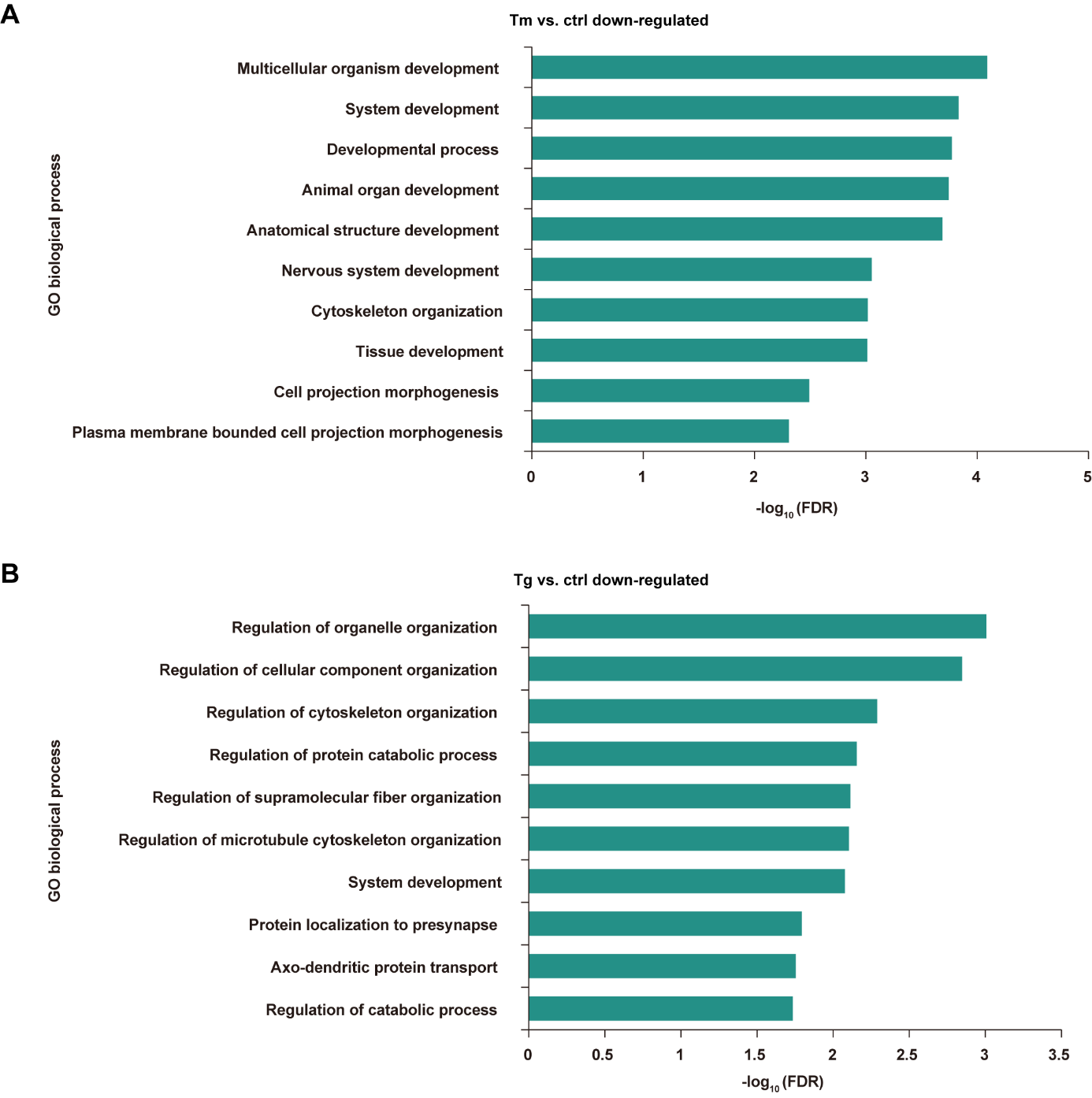
**Figure S4.** Gene ontology analysis of downregulated mRNAs from HEK293FT cells treated with A) tunicamycin or B) thapsigargin.


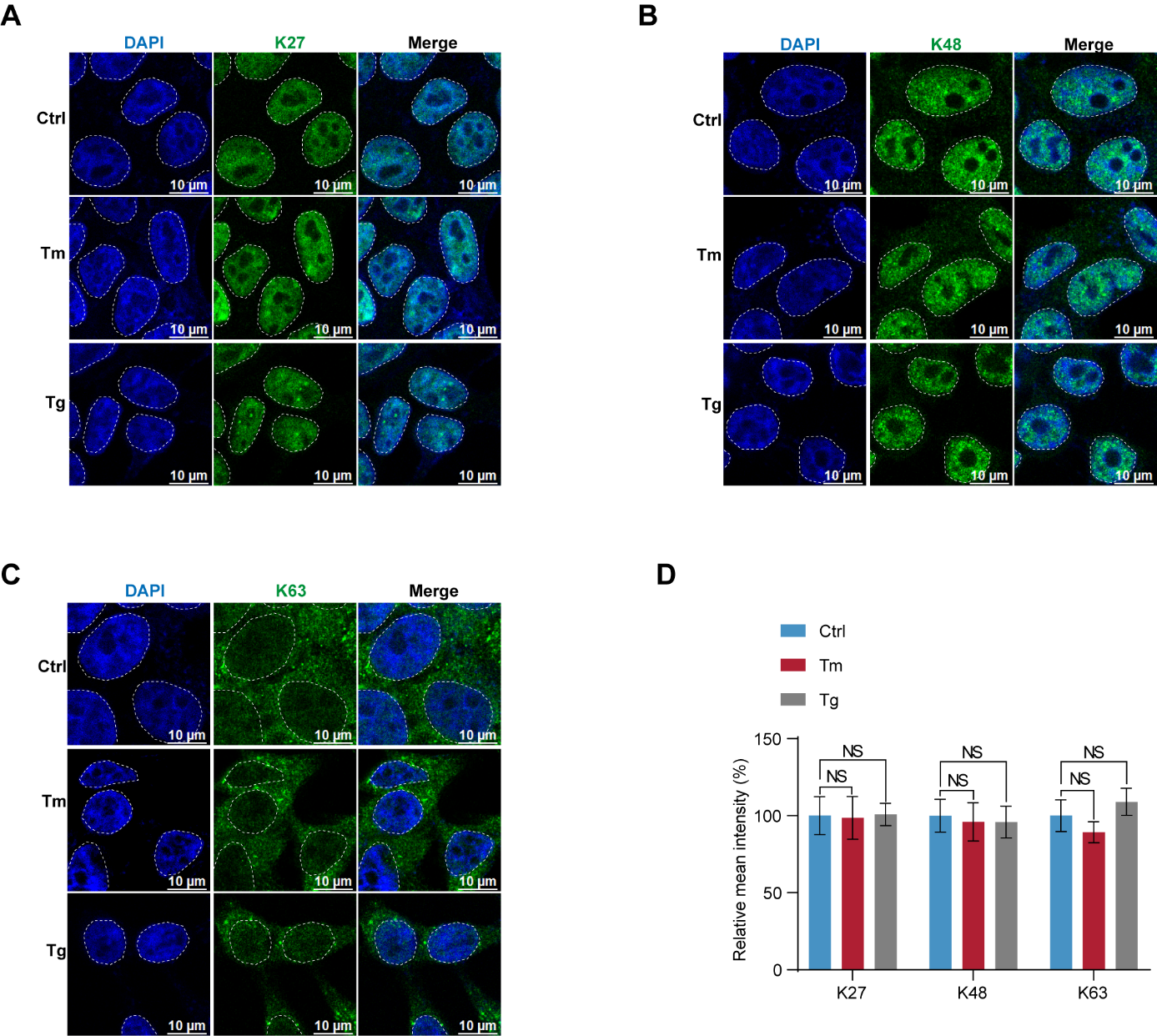
Figure S5. Distribution of K27-, K48-, and K63-linked ubiquitin chains in the nuclei. Representative immunofluorescence images showing the distribution of A) K27-linked ubiquitin chain, B) K48-linked ubiquitin chain and C) K63-linked ubiquitin chain in HEK293FT cells under induced (Tm, Tg) or untreated (Ctrl) conditions. The nuclei are outlined by white dashed lines. The scale bar represents 10 μm. D) Mean fluorescence intensity statistics are presented as the mean ± s.d. (n=100 cells, two-sided Student’s t test, ns p>0.05).


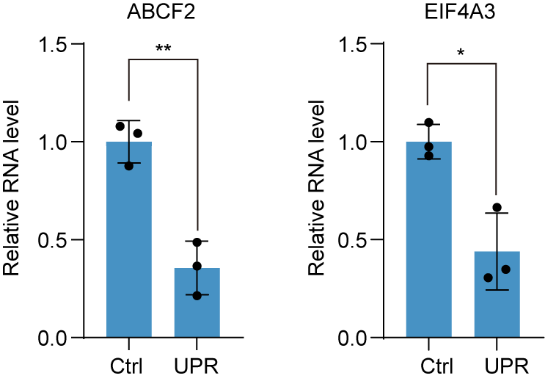


Figure S6. Relative mRNA levels of ABCF2 and EIF4A3 under induced (UPR) or untreated (Ctrl) conditions. The RPKM values of ABCF2 or ElF4A3 were calculated from RNA-seq data. (n= 3 biological replicates, mean ± s.d., two-sided Student’s t test, * p<0.05, ** p<0.01).


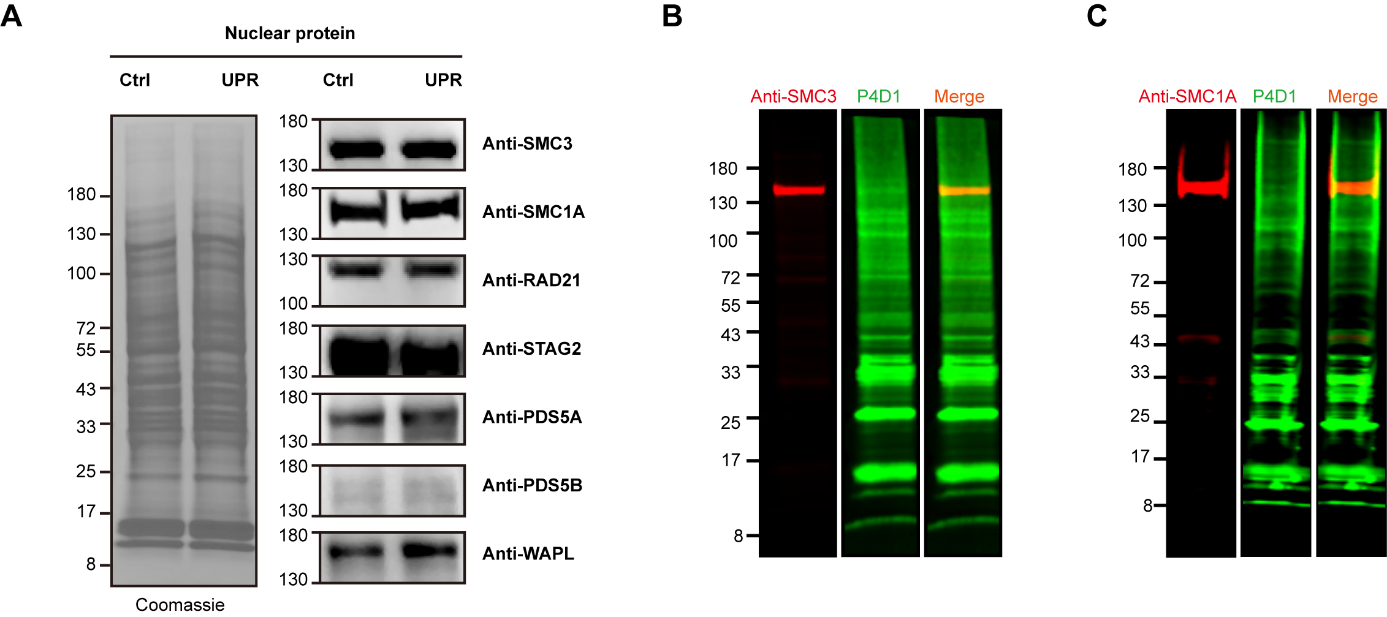
Figure S7. Cohesin is a target of K29-linked ubiquitination during the UPR. A) Coomassie blue-stained gel of nuclear proteins (left panel) under induced (UPR) or uninduced (Ctrl) conditions. In addition, parallel western blotting was performed with anti-SMC3, anti-SMC1A, anti-RAD21, anti-STAG2, anti-PDS5A, anti-PDS5B or anti-WAPL antibodies (right panel). B) Fluorescent western blotting was used to confirm the co-localization of SMC3 (anti-SMC3) and monoubiquitin (anti-monoubiquitin (P4D1)). C) Fluorescent western blotting was used to confirm the co-localization of SMC1A (anti-SMC1A) and monoubiquitin (anti-monoubiquitin (P4D1)).


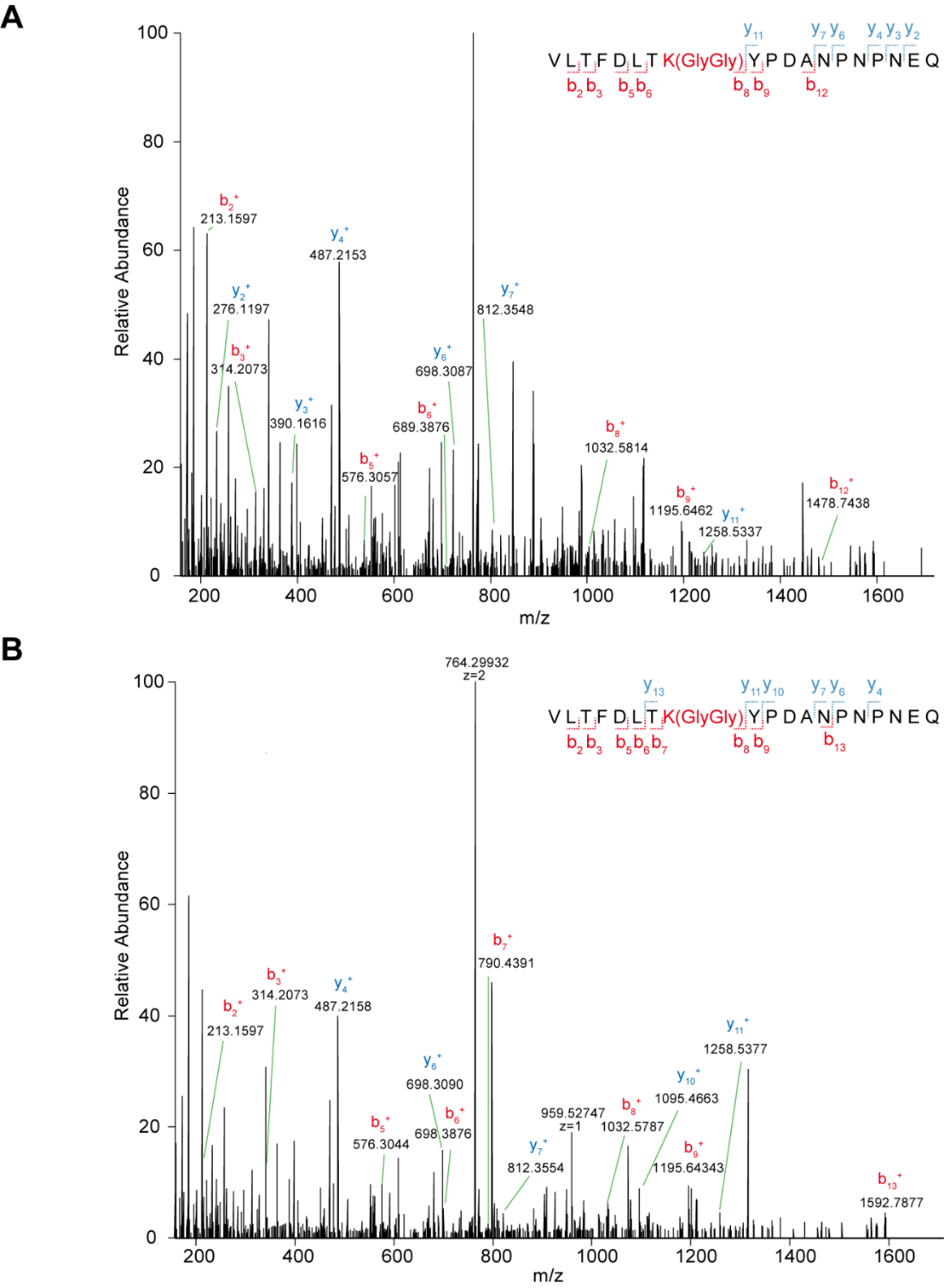


Figure S8. Peptide MS/MS spectra of SMC1A [1215-1233] under induced conditions.


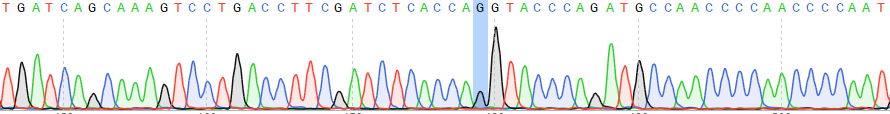


Figure S9. Verification of the SMC1A K1222R cell line. Sanger sequencing result of the target region. The blue region represents the mutated base.


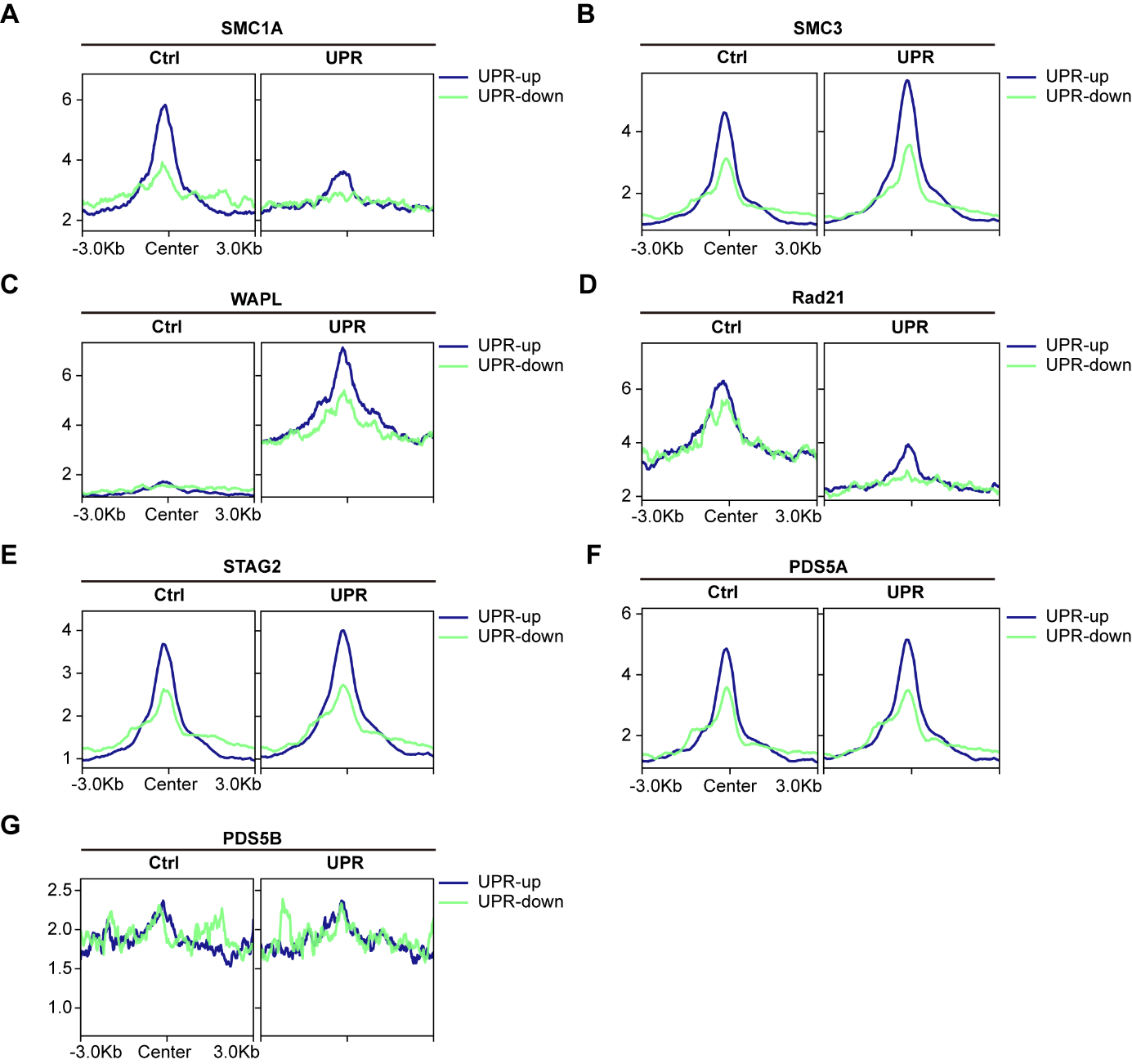
Figure S10. Line plots showing the relative CUT&Tag signal of A) SMC1A, B) SMC3, C) WAPL, D) RAD21, E) STAG2, F) PDS5A, and G) PDS5B in the loci of UPR-upregulated genes and UPR-downregulated genes.


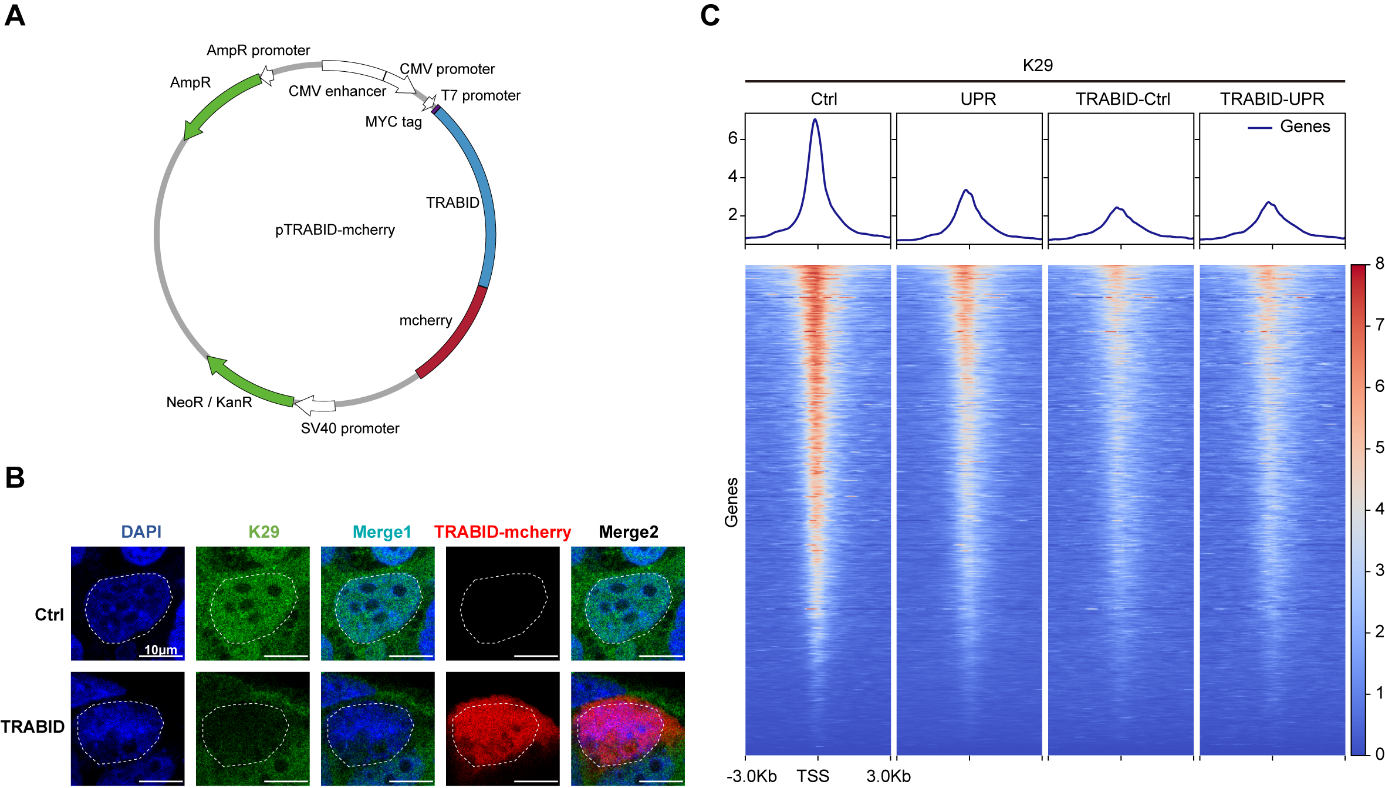
Figure S11. Construction of TRABID-OE-HEK293FT cells. A) Schematic diagram of the TRABID-mCherry plasmid for overexpression of the K29 deubiquitinase TRABID. B) Representative immunofluorescence images showing the distribution of K29-linked ubiquitin chains and TRABID-mCherry proteins in normal and TRABID-OE-HEK293FT cells. C) Total K29 CUT&TAG density heatmaps from TRABID-OE-HEK293FT cells (Tra) and WT-HEK293FT cells under induced (UPR) or untreated (Ctrl) conditions.


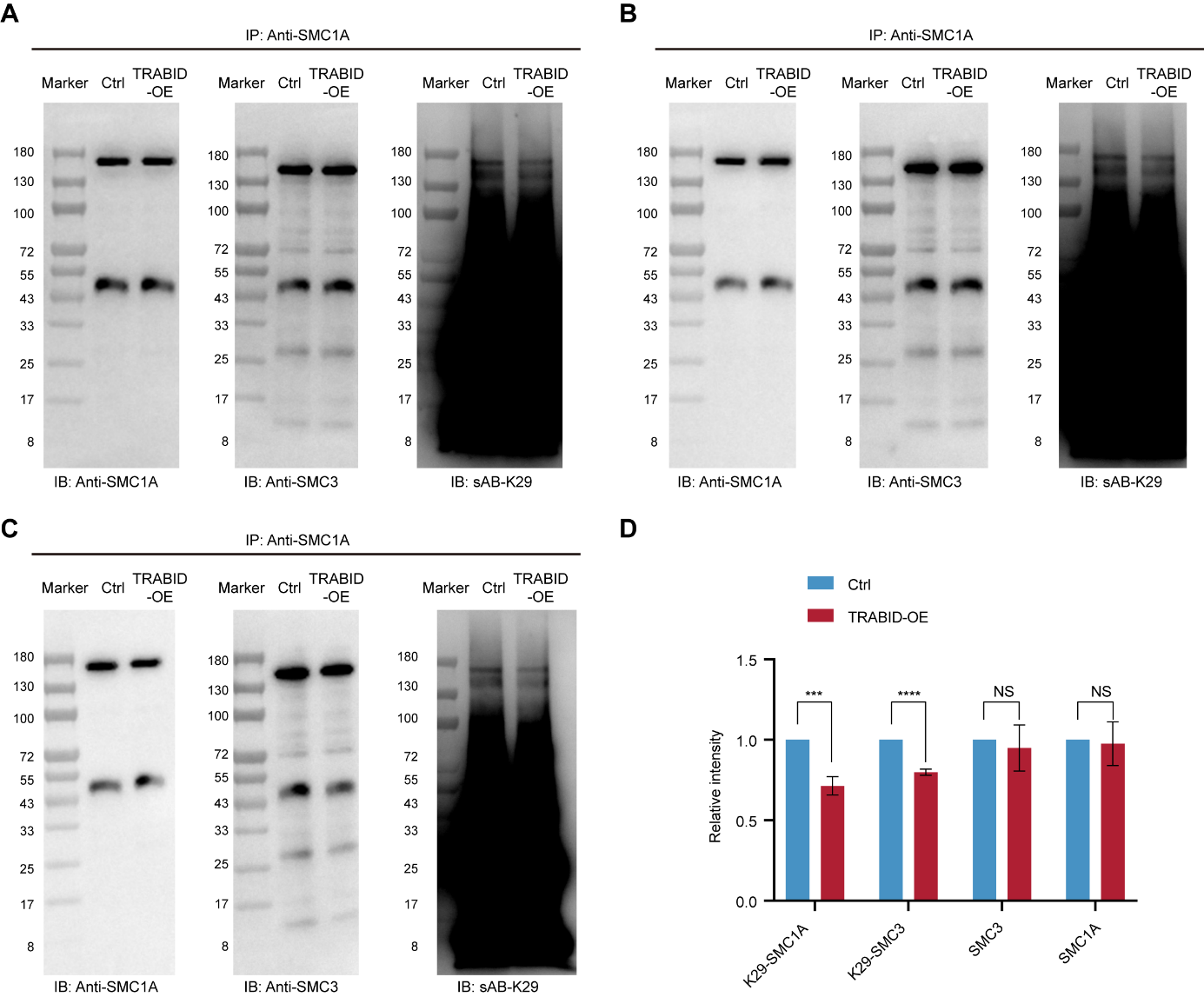


Figure S12. Downregulation of K29-linked ubiquitination of SMC1A / SMC3 in TRABID overexpression cells. A) B) C) The nuclear protein of Ctrl group and TRABID-OE group were extracted. Then Immunoprecipitation (IP) was performed using anti-SMC1A antibody, followed by western blotting (IB) using sAB-K29, anti-SMC1A or anti-SMC3 as the primary antibody. D) Relative intensity analysis of the SMC3 / SMC1A bands. (Data are presented as the means±s.d.s, n=3, two-sided Student’s t test, ns p>0.05, *** p<0.001, **** p<0.0001).


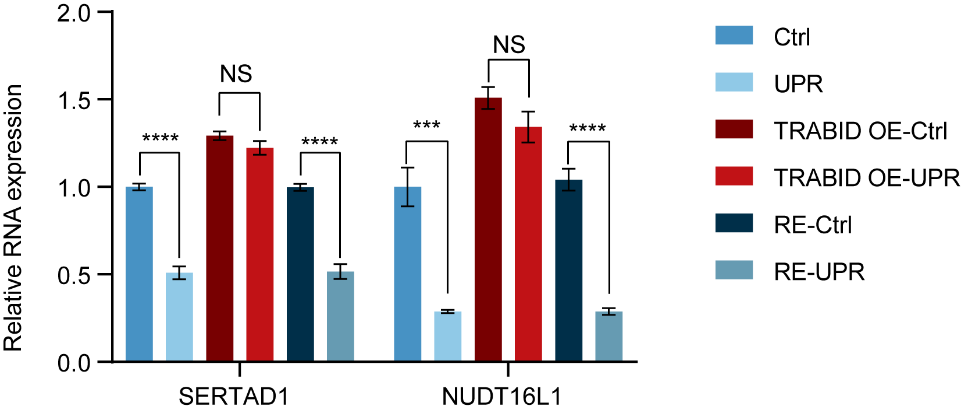


Figure S13. Rescue experiments of K29-depletion. The TRABID OE cells were cultured for 10 days to thoroughly remove the TRABID-plasmid (RE cells). The RNA was extracted and used for RT-qPCR. Data are presented as the means±s.d.s, n=3, two-sided Student’s t test, ns p>0.05, *** p<0.001, **** p<0.0001).


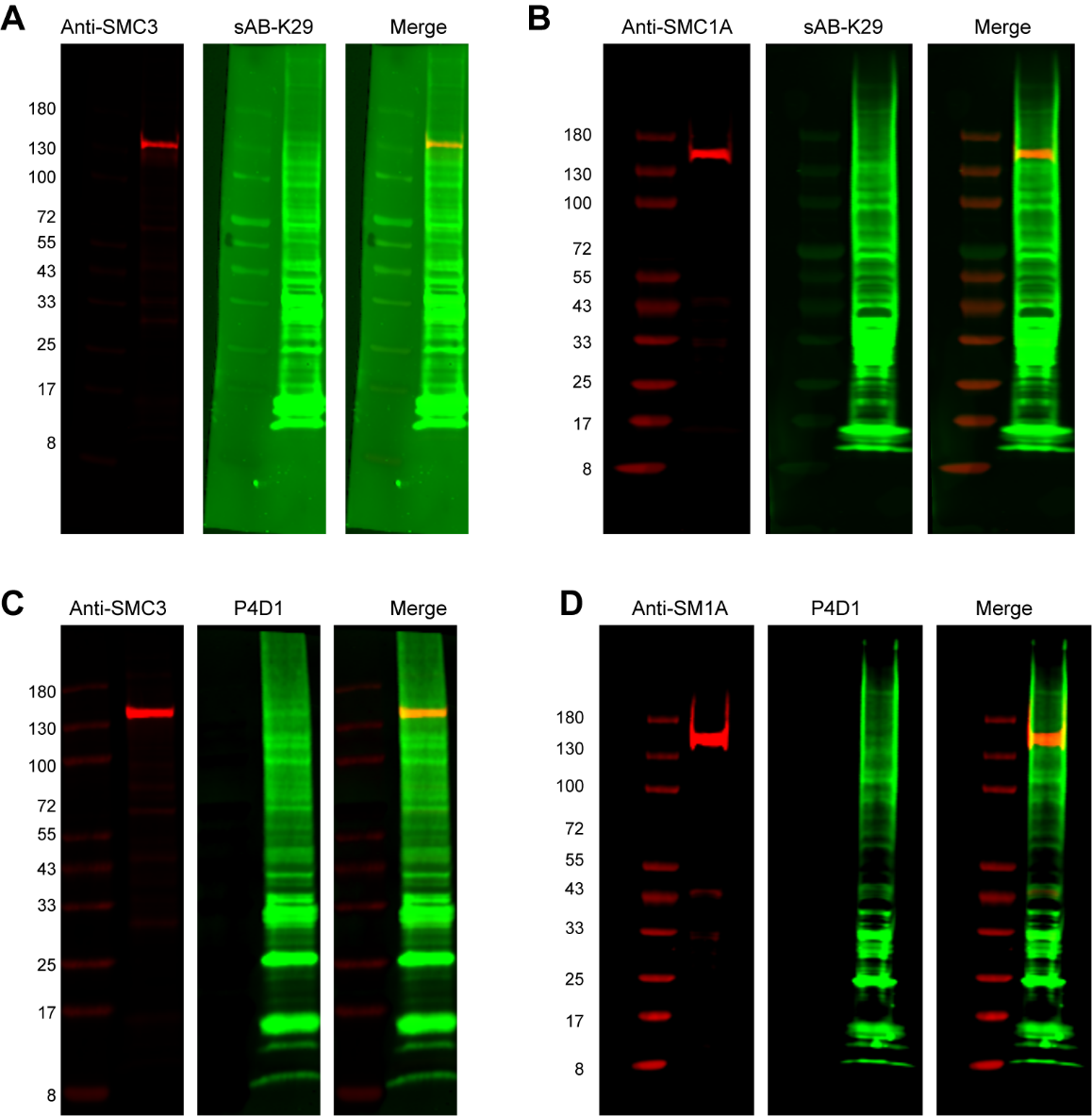


Figure S14. The uncropped gel images of fluorescent western blotting analysis. A) Uncropped gel image for Figure 4A. B) Uncropped gel image for Figure 4B. C) Uncropped gel image for Figure S7B. D) Uncropped gel image for Figure S7C.


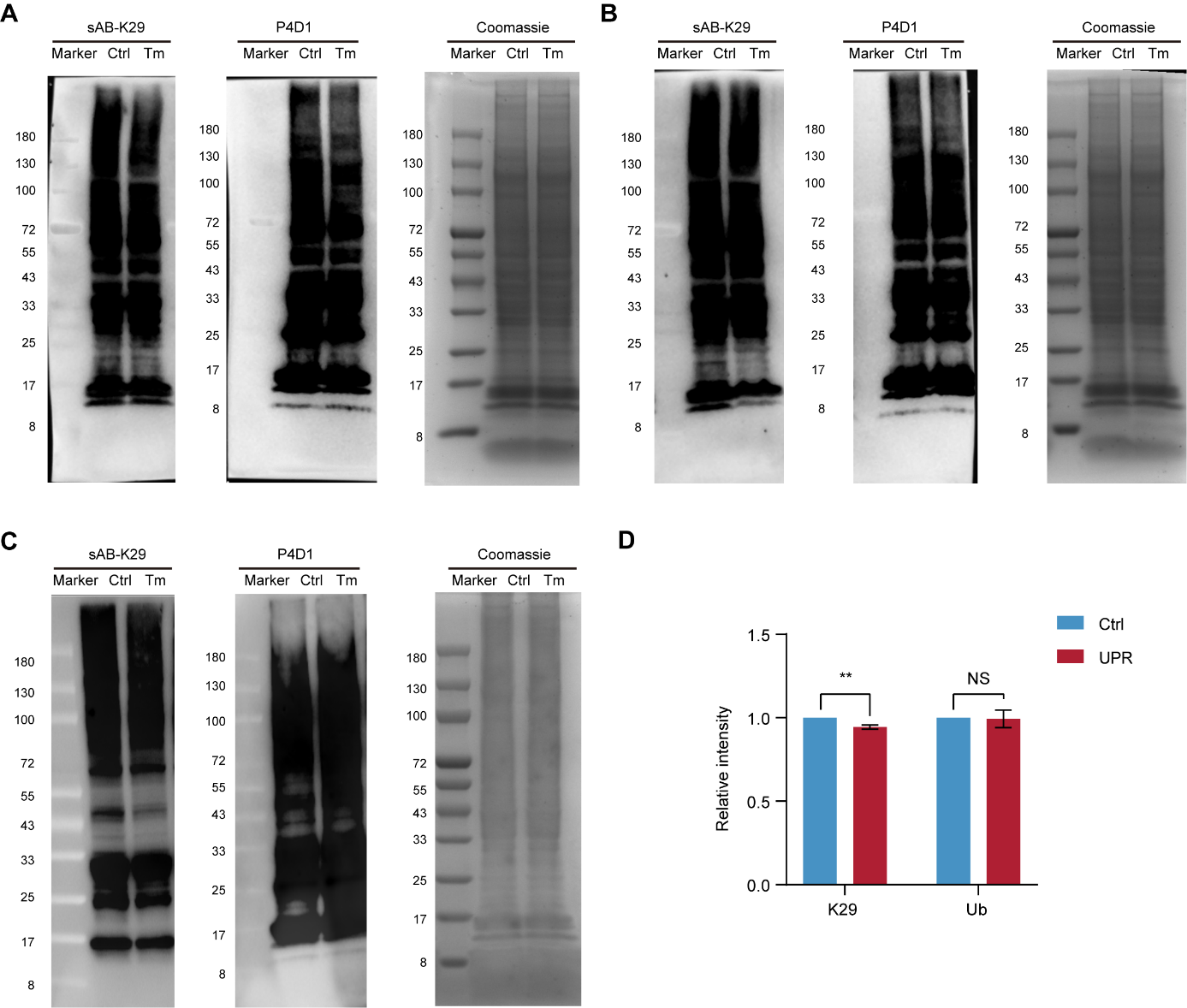
Figure S15. The uncropped gel images of western blotting analysis for Figure 2. A) Uncropped gel image for Figure 2D. B) C) The other two repeats for statistical tests. D) Relative intensity analysis of the bands. (Data are presented as the means±s.d.s, n=3, two-sided Student’s t test, ns p>0.05, ** p<0.01).


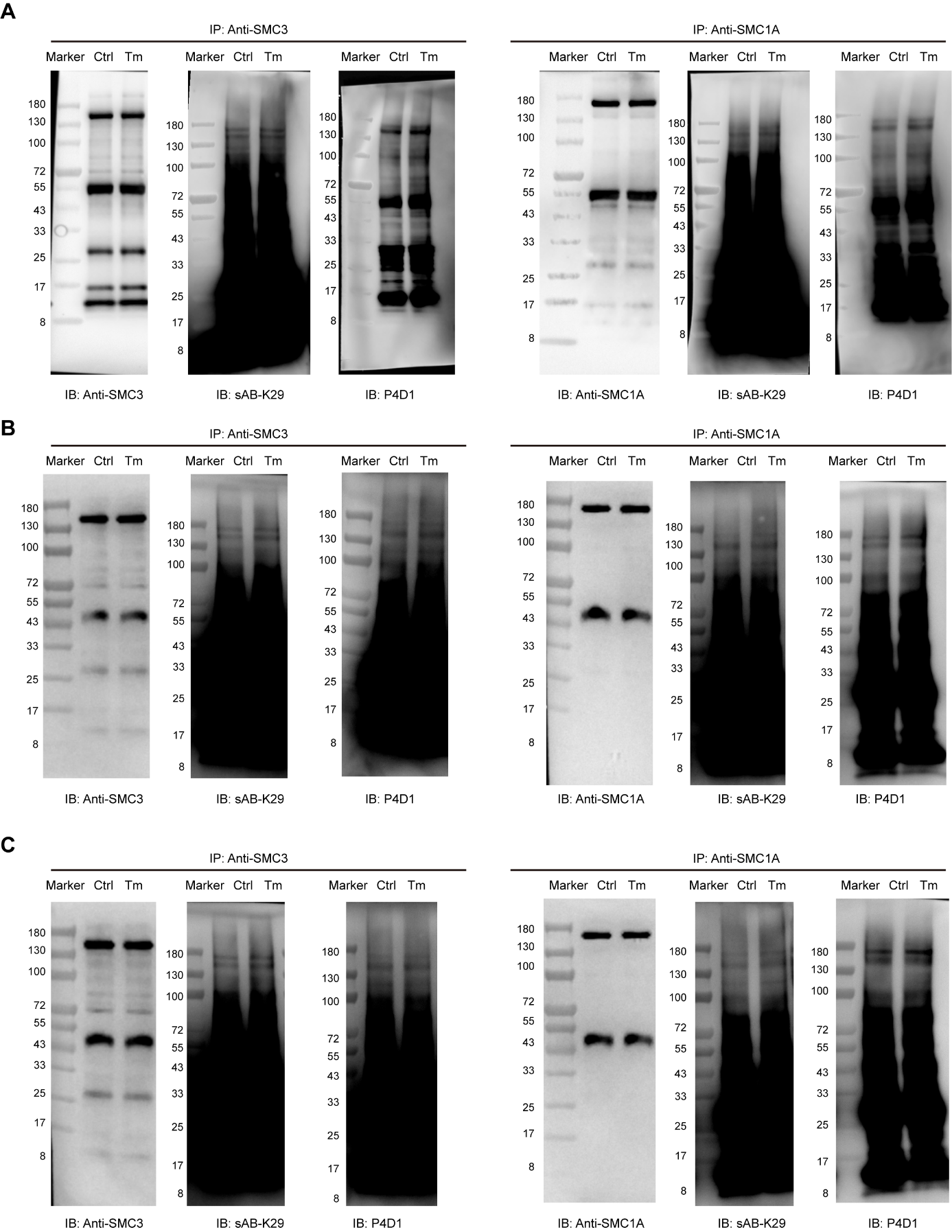
Figure S16. The uncropped gel images of western blotting analysis for Figure 4. A) Uncropped gel image for Figure 4C and 4E. B) C) The other two repeats for statistical tests in Figure 4D and 4F.


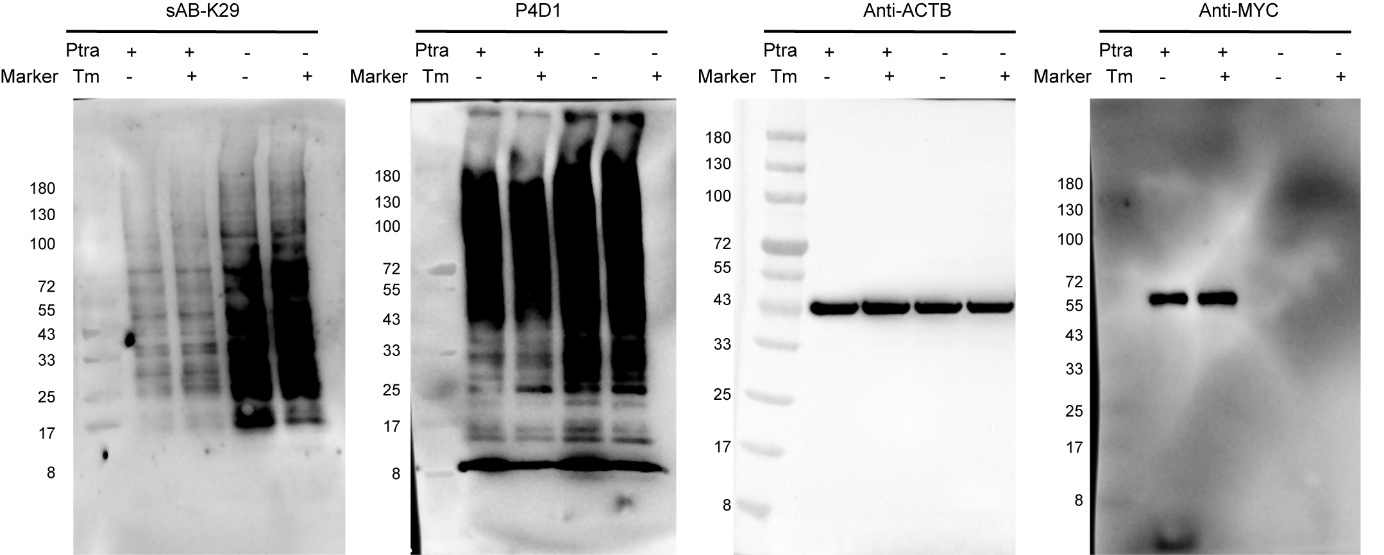
Figure S17. The uncropped gel images of western blotting analysis for Figure 5A**.**


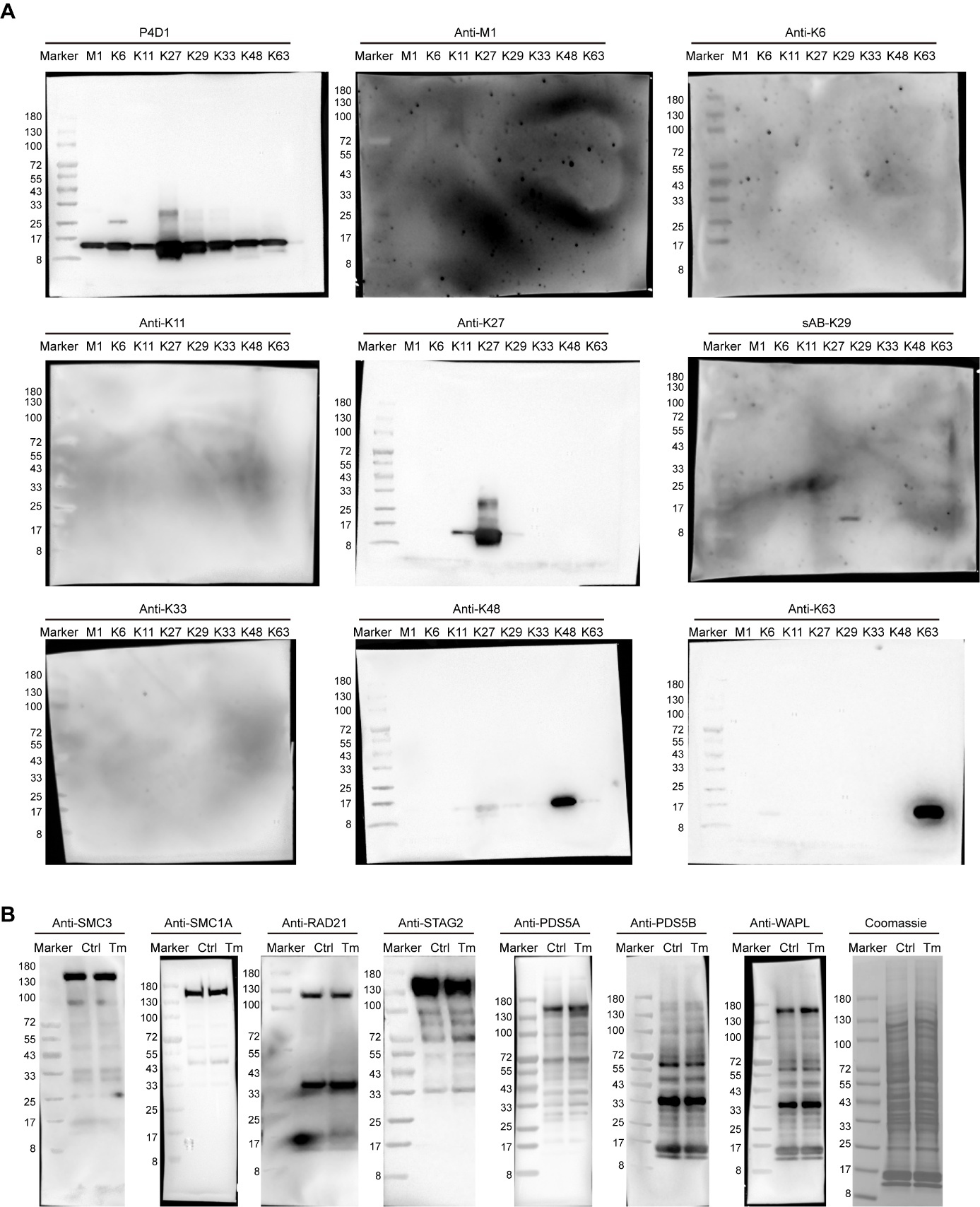
Figure S18. The uncropped gel images of western blotting analysis for Figure S1 and S7. A) Uncropped gel image for Figure S1. B) Uncropped gel image for Figure S7A.

**Table S1.** 37 predicted transcription factors by LISA.

| KDM4A | NR2F1 | ESR1 | MBD2 |
| --- | --- | --- | --- |
| GLIS1 | SMC3 | NEUROD1 | EGR3 |
| TCF7L1 | E2F6 | CNOT3 | CTCF |
| ERG | JMJD6 | 552-SKD | SUZ12 |
| IKZF2 | MYH11 | TFAP2C | SMC1A |
| EGR2 | RAD21 | MYC | SRF |
| CBFB | SP1 | SP2 | TFAP2A |
| MAX | ZBTB33 | TRIM25 |  |
| 598-SKD | KDM2B | KLF9 |  |
| KLF15 | EZH2 | SP4 |  |

**Table S2.** Plasmid sequences used in this work

| **Plasmid** | **Sequence** |
| --- | --- |
| pTUBEs (For generating TUBEs) | ATACACTCCGCTATCGCTACGTGACTGGGTCATGGCTGCGCCCCGACACCCGCCAACACCCGCTGACGCGCCCTGACGGGCTTGTCTGCTCCCGGCATCCGCTTACAGACAAGCTGTGACCGTCTCCGGGAGCTGCATGTGTCAGAGGTTTTCACCGTCATCACCGAAACGCGCGAGGCAGCTGCGGTAAAGCTCATCAGCGTGGTCGTGCAGCGATTCACAGATGTCTGCCTGTTCATCCGCGTCCAGCTCGTTGAGTTTCTCCAGAAGCGTTAATGTCTGGCTTCTGATAAAGCGGGCCATGTTAAGGGCGGTTTTTTCCTGTTTGGTCACTGATGCCTCCGTGTAAGGGGGATTTCTGTTCATGGGGGTAATGATACCGATGAAACGAGAGAGGATGCTCACGATACGGGTTACTGATGATGAACATGCCCGGTTACTGGAACGTTGTGAGGGTAAACAACTGGCGGTATGGATGCGGCGGGACCAGAGAAAAATCACTCAGGGTCAATGCCAGCCGAACGCCAGCAAGACGTAGCCCAGCGCGTCGGCCGCCATGCCGGCGATAATGGCCTGCTTCTCGCCGAAACGTTTGGTGGCGGGACCAGTGACGAAGGCTTGAGCGAGGGCGTGCAAGATTCCGAATACCGCAAGCGACAGGCCGATCATCGTCGCGCTCCAGCGAAAGCGGTCCTCGCCGAAAATGACCCAGAGCGCTGCCGGCACCTGTCCTACGAGTTGCATGATAAAGAAGACAGTCATAAGTGCGGCGACGATAGTCATGCCCCGCGCCCACCGGAAGGAGCTGACTGGGTTGAAGGCTCTCAAGGGCATCGGTCGAGATCCCGGTGCCTAATGAGTGAGCTAACTTACATTAATTGCGTTGCGCTCACTGCCCGCTTTCCAGTCGGGAAACCTGTCGTGCCAGCTGCATTAATGAATCGGCCAACGCGCGGGGAGAGGCGGTTTGCGTATTGGGCGCCAGGGTGGTTTTTCTTTTCACCAGTGAGACGGGCAACAGCTGATTGCCCTTCACCGCCTGGCCCTGAGAGAGTTGCAGCAAGCGGTCCACGCTGGTTTGCCCCAGCAGGCGAAAATCCTGTTTGATGGTGGTTAACGGCGGGATATAACATGAGCTGTCTTCGGTATCGTCGTATCCCACTACCGAGATATCCGCACCAACGCGCAGCCCGGACTCGGTAATGGCGCGCATTGCGCCCAGCGCCATCTGATCGTTGGCAACCAGCATCGCAGTGGGAACGATGCCCTCATTCAGCATTTGCATGGTTTGTTGAAAACCGGACATGGCACTCCAGTCGCCTTCCCGTTCCGCTATCGGCTGAATTTGATTGCGAGTGAGATATTTATGCCAGCCAGCCAGACGCAGACGCGCCGAGACAGAACTTAATGGGCCCGCTAACAGCGCGATTTGCTGGTGACCCAATGCGACCAGATGCTCCACGCCCAGTCGCGTACCGTCTTCATGGGAGAAAATAATACTGTTGATGGGTGTCTGGTCAGAGACATCAAGAAATAACGCCGGAACATTAGTGCAGGCAGCTTCCACAGCAATGGCATCCTGGTCATCCAGCGGATAGTTAATGATCAGCCCACTGACGCGTTGCGCGAGAAGATTGTGCACCGCCGCTTTACAGGCTTCGACGCCGCTTCGTTCTACCATCGACACCACCACGCTGGCACCCAGTTGATCGGCGCGAGATTTAATCGCCGCGACAATTTGCGACGGCGCGTGCAGGGCCAGACTGGAGGTGGCAACGCCAATCAGCAACGACTGTTTGCCCGCCAGTTGTTGTGCCACGCGGTTGGGAATGTAATTCAGCTCCGCCATCGCCGCTTCCACTTTTTCCCGCGTTTTCGCAGAAACGTGGCTGGCCTGGTTCACCACGCGGGAAACGGTCTGATAAGAGACACCGGCATACTCTGCGACATCGTATAACGTTACTGGTTTCACATTCACCACCCTGAATTGACTCTCTTCCGGGCGCTATCATGCCATACCGCGAAAGGTTTTGCGCCATTCGATGGTGTCCGGGATCTCGACGCTCTCCCTTATGCGACTCCTGCATTAGGAAGCAGCCCAGTAGTAGGTTGAGGCCGTTGAGCACCGCCGCCGCAAGGAATGGTGCATGCCGGCATGCCGCCCTTTCGTCTTCAAGAATTAATTCCCAATTCCCCAGGCATCAAATAAAACGAAAGGCTCAGTCGAAAGACTGGGCCTTTCGTTTTATCTGTTGTTTGTCGGTGAACGCTCTCCTGAGTAGGACAAATCCGCCGGGAGCGGATTTGAACGTTGCGAAGCAACGGCCCGGAGGGTGGCGGGCAGGACGCCCGCCATAAACTGCCAGGAATTAATTCCCCAGGCATCAAATAAAACGAAAGGCTCAGTCGAAAGACTGGGCCTTTCGTTTTATCTGTTGTTTGTCGGTGAACGCTCTCCTGAGTAGGACAAATCCGCCGGGAGCGGATTTGAACGTTGCGAAGCAACGGCCCGGAGGGTGGCGGGCAGGACGCCCGCCATAAACTGCCAGGAATTAATTCCCCAGGCATCAAATAAAACGAAAGGCTCAGTCGAAAGACTGGGCCTTTCGTTTTATCTGTTGTTTGTCGGTGAACGCTCTCCTGAGTAGGACAAATCCGCCGGGAGCGGATTTGAACGTTGCGAAGCAACGGCCCGGAGGGTGGCGGGCAGGACGCCCGCCATAAACTGCCAGGAATTAATTCCCCAGGCATCAAATAAAACGAAAGGCTCAGTCGAAAGACTGGGCCTTTCGTTTTATCTGTTGTTTGTCGGTGAACGCTCTCCTGAGTAGGACAAATCCGCCGGGAGCGGATTTGAACGTTGCGAAGCAACGGCCCGGAGGGTGGCGGGCAGGACGCCCGCCATAAACTGCCAGGAATTAATTCCCCAGGCATCAAATAAAACGAAAGGCTCAGTCGAAAGACTGGGCCTTTCGTTTTATCTGTTGTTTGTCGGTGAACGCTCTCCTGAGTAGGACAAATCCGCCGGGAGCGGATTTGAACGTTGCGAAGCAACGGCCCGGAGGGTGGCGGGCAGGACGCCCGCCATAAACTGCCAGGAATTGGGGATCGGAATTAATTCCCGGTTTAAACCGGGGATCTCGATCCCGCGAAATTAATACGACTCACTATAGGGGAATTGTGAGCGGATAACAATTCCCCTCTAGAAATAATTTTGTTTAACTTTAAGAAGGAGATATACCATGGGTGATTACAAGGATCACGATGGCGATTACAAGGATCACGATATCGATTACAAGGATGATGATGATAAGATGACCATGATTACGCCAAGCTTAATGGCCAGCATGACCGGCGGTCAGCAGATGGGCGATATTGGTGGCGGCGGCAGTGGTGGCGGCGTGAATCCTCAGCTGCAGAATCCGGAAGTTGCCTTTCAGCAGCAGCTGGAACAGCTGAGCGCCATGGGTTTTCTGAATGCAGAAGCAAATCTGCAGGCACTGATTGCAACCGGCGGCGATATTAATGCCGCAATTGAAGCACTGCTGGGTAGCCAGCCGAGCGGTGGCGGTGGTAGTGGCGGTGGTGTTAATCCGCAGCTGCAGAACCCGGAAGTGGCCTTTCAGCAACAGCTGGAACAACTGAGTGCAATGGGTTTTCTTAATGCAGAAGCGAATCTGCAGGCCCTGATTGCCACCGGTGGTGACATTAATGCAGCCATTGAAGCCCTGCTGGGCAGCCAGCCGTCAGGTGGTGGTGGTAGCGGTGGCGGCGTTAATCCGCAACTGCAGAATCCTGAAGTGGCATTTCAGCAGCAACTGGAACAGTTAAGTGCCATGGGTTTCCTGAATGCAGAGGCCAATCTGCAGGCTCTGATTGCCACAGGCGGTGACATTAACGCCGCAATTGAGGCCCTGCTGGGTTCACAGCCGAGTGGCGGCGGTGGTAGCGGCGGTGGTGTGAATCCGCAGTTACAGAATCCGGAGGTTGCCTTTCAACAGCAGCTGGAGCAGCTGAGCGCAATGGGCTTTCTGAATGCCGAAGCAAATCTTCAGGCCCTGATCGCAACCGGTGGTGACATCAATGCAGCCATCGAAGCACTGTTAGGCAGTCAGCCGAGTGGTGGTGGTGGCAGCGGTGGTGGCGTGAATCCGCAATTACAGAATCCTGAGGTGGCCTTTCAACAACAGCTGGAGCAACTGAGCGCCATGGGCTTTTTAAATGCCGAAGCCAATCTGCAAGCCCTGATTGCGACCGGTGGCGATATTAACGCCGCCATTGAAGCGCTGCTGGGTAGTCAGCCGTCAGGCGGCGGCGGTAGTGGTGGTGGAGTTAATCCGCAGCTTCAGAATCCGGAAGTGGCGTTTCAGCAGCAGTTAGAACAGCTGAGTGCAATGGGCTTCCTGAATGCCGAGGCCAATCTTCAGGCACTGATCGCAACAGGTGGTGACATAAATGCAGCAATTGAAGCGTTACTGGGTAGCCAACCGAGTGGCGGTGGCGGCAGTCTGCCGGAAACCGGTGGTTGCATCACGGGAGATGCACTAGTTGCCCTACCCGAGGGCGAGTCGGTACGCATCGCCGACATCGTGCCGGGTGCGCGGCCCAACAGTGACAACGCCATCGACCTGAAAGTCCTTGACCGGCATGGCAATCCCGTGCTCGCCGACCGGCTGTTCCACTCCGGCGAGCATCCGGTGTACACGGTGCGTACGGTCGAAGGTCTGCGTGTGACGGGCACCGCGAACCACCCGTTGTTGTGTTTGGTCGACGTCGCCGGGGTGCCGACCCTGCTGTGGAAGCTGATCGACGAAATCAAGCCGGGCGATTACGCGGTGATTCAACGCAGCGCATTCAGCGTCGACTGTGCAGGTTTTGCCCGCGGGAAACCCGAATTTGCGCCCACAACCTACACAGTCGGCGTCCCTGGACTGGTGCGTTTCTTGGAAGCACACCACCGAGACCCGGACGCCCAAGCTATCGCCGACGAGCTGACCGACGGGCGGTTCTACTACGCGAAAGTCGCCAGTGTCACCGACGCCGGCGTGCAGCCGGTGTATAGCCTTCGTGTCGACACGGCAGACCACGCGTTTATCACGAACGGGTTCGTCAGCCACGCTACTGGCCTCACCGGTCTGAACTCAGGCCTCACGACAAATCCTGGTGTATCCGCTTGGCAGGTCAACACAGCTTATACTGCGGGACAATTGGTCACATATAACGGCAAGACGTATAAATGTTTGCAGCCCCACACCTCCTTGGCAGGATGGGAACCATCCAACGTTCCTGCCTTGTGGCAGCTTCAATGACTGCAGGAAGGGGATCCGGCTGCTAACAAAGCCCGAAAGGAAGCTGAGTTGGCTGCTGCCACCGCTGAGCAATAACTAGCATAACCCCTTGGGGCCTCTAAACGGGTCTTGAGGGGTTTTTTGCTGAAAGGAGGAACTATATCCGGATAACTACGTCAGGTGGCACTTTTCGGGGAAATGTGCGCGGAACCCCTATTTGTTTATTTTTCTAAATACATTCAAATATGTATCCGCTCATGAGACAATAACCCTGATAAATGCTTCAATAATATTGAAAAAGGAAGAGTATGAGTATTCAACATTTCCGTGTCGCCCTTATTCCCTTTTTTGCGGCATTTTGCCTTCCTGTTTTTGCTCACCCAGAAACGCTGGTGAAAGTAAAAGATGCTGAAGATCAGTTGGGTGCACGAGTGGGTTACATCGAACTGGATCTCAACAGCGGTAAGATCCTTGAGAGTTTTCGCCCCGAAGAACGTTTCCCAATGATGAGCACTTTTAAAGTTCTGCTATGTGGCGCGGTATTATCCCGTGTTGACGCCGGGCAAGAGCAACTCGGTCGCCGCATACACTATTCTCAGAATGACTTGGTTGAGTACTCACCAGTCACAGAAAAGCATCTTACGGATGGCATGACAGTAAGAGAATTATGCAGTGCTGCCATAACCATGAGTGATAACACTGCGGCCAACTTACTTCTGACAACGATCGGAGGACCGAAGGAGCTAACCGCTTTTTTGCACAACATGGGGGATCATGTAACTCGCCTTGATCGTTGGGAACCGGAGCTGAATGAAGCCATACCAAACGACGAGCGTGACACCACGATGCCTGTAGCAATGGCAACAACGTTGCGCAAACTATTAACTGGCGAACTACTTACTCTAGCTTCCCGGCAACAATTAATAGACTGGATGGAGGCGGATAAAGTTGCAGGACCACTTCTGCGCTCGGCCCTTCCGGCTGGCTGGTTTATTGCTGATAAATCTGGAGCCGGTGAGCGTGGGTCTCGCGGTATCATTGCAGCACTGGGGCCAGATGGTAAGCCCTCCCGTATCGTAGTTATCTACACGACGGGGAGTCAGGCAACTATGGATGAACGAAATAGACAGATCGCTGAGATAGGTGCCTCACTGATTAAGCATTGGTAACTGTCAGACCAAGTTTACTCATATATACTTTAGATTGATTTACCCCGGTTGATAATCAGAAAAGCCCCAAAAACAGGAAGATTGTATAAGCAAATATTTAAATTGTAAACGTTAATATTTTGTTAAAATTCGCGTTAAATTTTTGTTAAATCAGCTCATTTTTTAACCAATAGGCCGAAATCGGCAAAATCCCTTATAAATCAAAAGAATAGCCCGAGATAGGGTTGAGTGTTGTTCCAGTTTGGAACAAGAGTCCACTATTAAAGAACGTGGACTCCAACGTCAAAGGGCGAAAAACCGTCTATCAGGGCGATGGCCCACTACGTGAACCATCACCCAAATCAAGTTTTTTGGGGTCGAGGTGCCGTAAAGCACTAAATCGGAACCCTAAAGGGAGCCCCCGATTTAGAGCTTGACGGGGAAAGCCGGCGAACGTGGCGAGAAAGGAAGGGAAGAAAGCGAAAGGAGCGGGCGCTAGGGCGCTGGCAAGTGTAGCGGTCACGCTGCGCGTAACCACCACACCCGCCGCGCTTAATGCGCCGCTACAGGGCGCGTAAAAGGATCTAGGTGAAGATCCTTTTTGATAATCTCATGACCAAAATCCCTTAACGTGAGTTTTCGTTCCACTGAGCGTCAGACCCCGTAGAAAAGATCAAAGGATCTTCTTGAGATCCTTTTTTTCTGCGCGTAATCTGCTGCTTGCAAACAAAAAAACCACCGCTACCAGCGGTGGTTTGTTTGCCGGATCAAGAGCTACCAACTCTTTTTCCGAAGGTAACTGGCTTCAGCAGAGCGCAGATACCAAATACTGTCCTTCTAGTGTAGCCGTAGTTAGGCCACCACTTCAAGAACTCTGTAGCACCGCCTACATACCTCGCTCTGCTAATCCTGTTACCAGTGGCTGCTGCCAGTGGCGATAAGTCGTGTCTTACCGGGTTGGACTCAAGACGATAGTTACCGGATAAGGCGCAGCGGTCGGGCTGAACGGGGGGTTCGTGCACACAGCCCAGCTTGGAGCGAACGACCTACACCGAACTGAGATACCTACAGCGTGAGCTATGAGAAAGCGCCACGCTTCCCGAAGGGAGAAAGGCGGACAGGTATCCGGTAAGCGGCAGGGTCGGAACAGGAGAGCGCACGAGGGAGCTTCCAGGGGGAAACGCCTGGTATCTTTATAGTCCTGTCGGGTTTCGCCACCTCTGACTTGAGCGTCGATTTTTGTGATGCTCGTCAGGGGGGCGGAGCCTATGGAAAAACGCCAGCAACGCGGCCTTTTTACGGTTCCTGGCCTTTTGCTGGCCTTTTGCTCACATGTTCTTTCCTGCGTTATCCCCTGATTCTGTGGATAACCGTATTACCGCCTTTGAGTGAGCTGATACCGCTCGCCGCAGCCGAACGACCGAGCGCAGCGAGTCAGTGAGCGAGGAAGCTATGGTGCACTCTCAGTACAATCTGCTCTGATGCCGCATAGTTAAGCCAGT |
| pTRABID-mcherry (For generating K29-depletion cells) | GACGGATCGGGAGATCTCCCGATCCCCTATGGTGCACTCTCAGTACAATCTGCTCTGATGCCGCATAGTTAAGCCAGTATCTGCTCCCTGCTTGTGTGTTGGAGGTCGCTGAGTAGTGCGCGAGCAAAATTTAAGCTACAACAAGGCAAGGCTTGACCGACAATTGCATGAAGAATCTGCTTAGGGTTAGGCGTTTTGCGCTGCTTCGCGATGTACGGGCCAGATATACGCGTTGACATTGATTATTGACTAGTTATTAATAGTAATCAATTACGGGGTCATTAGTTCATAGCCCATATATGGAGTTCCGCGTTACATAACTTACGGTAAATGGCCCGCCTGGCTGACCGCCCAACGACCCCCGCCCATTGACGTCAATAATGACGTATGTTCCCATAGTAACGCCAATAGGGACTTTCCATTGACGTCAATGGGTGGAGTATTTACGGTAAACTGCCCACTTGGCAGTACATCAAGTGTATCATATGCCAAGTACGCCCCCTATTGACGTCAATGACGGTAAATGGCCCGCCTGGCATTATGCCCAGTACATGACCTTATGGGACTTTCCTACTTGGCAGTACATCTACGTATTAGTCATCGCTATTACCATGGTGATGCGGTTTTGGCAGTACATCAATGGGCGTGGATAGCGGTTTGACTCACGGGGATTTCCAAGTCTCCACCCCATTGACGTCAATGGGAGTTTGTTTTGGCACCAAAATCAACGGGACTTTCCAAAATGTCGTAACAACTCCGCCCCATTGACGCAAATGGGCGGTAGGCGTGTACGGTGGGAGGTCTATATAAGCAGAGCTCTCTGGCTAACTAGAGAACCCACTGCTTACTGGCTTATCGAAATTAATACGACTCACTATAGGGAGACCCAAGCTGGCTAGCATGGAGCAAAAGCTCATTTCTGAAGAGGACTTGCTGGAAGTGGACTTTAAAAAACTGAAGCAAATTAAAAACAGGATGAAAAAGACTGATTGGCTCTTCCTCAATGCTTGTGTGGGGGTTGTGGAAGGTGATCTGGCTGCCATAGAAGCATACAAGTCATCAGGAGGAGACATTGCAAGGCAGCTCACCGCAGATGAAGTGCGCCTGCTGAATAGGCCTTCTGCCTTTGATGTTGGCTATACTCTGGTGCACCTGGCTATAAGGTTTCAGAGGCAGGATATGCTGGCAATACTGCTGACAGAGGTGTCTCAACAAGCAGCAAAGTGTATTCCAGCAATGGTGTGTCCTGAACTGACAGAACAAATCCGGAGAGAGATAGCTGCCTCTCTGCATCAGAGAAAGGGGGATTTTGCTTGCTATTTTCTGACTGACCTGGTGACATTTACACTGCCAGCAGATATTGAAGATCTGCCCCCAACAGTCCAAGAAAAACTGTTTGATGAGGTGCTGGATAGAGACGTTCAAAAAGAACTGGAAGAAGAATCTCCAATTATTAACTGGTCCCTGGAACTGGCTACAAGGCTGGACAGTAGGCTGTATGCACTGTGGAACCGGACTGCAGGAGACTGCCTGCTGGATTCAGTTCTGCAAGCTACCTGGGGCATCTATGACAAGGACTCAGTGCTGCGGAAAGCCCTGCATGACAGCCTGCATGACTGTTCACATTGGTTTTACACACGCTGGAAAGATTGGGAATCATGGTATTCTCAGAGCTTTGGTCTGCATTTTTCCCTGAGAGAAGAACAGTGGCAAGAAGACTGGGCATTTATACTCTCTCTGGCTAGTCAGCCTGGAGCAAGCCTGGAGCAGACCCACATTTTTGTGCTGGCACATATTCTGAGAAGGCCAATTATAGTTTATGGAGTGAAATATTACAAGAGTTTCCGGGGAGAAACTCTGGGATATACTCGGTTTCAAGGTGTTTATCTGCCTCTGCTGTGGGAACAGAGTTTTTGTTGGAAAAGTCCCATTGCTCTGGGTTATACCAGGGGCCACTTCTCTGCTCTGGTTGCCATGGAAAATGATGGCTATGGCAACAGGGGTGCTGGTGCTAATCTCAATACCGATGATGATGTCACCATCACATTTCTGCCTCTGGTTGACAGTGAAAGGAAGCTGCTCCATGTGCACTTCCTGTCTGCTCAGGAGCTGGGTAATGAGGAACAGCAAGAAAAACTGCTCAGGGAGTGGCTGGACTGCTGTGTGACCGAGGGGGGAGTTCTGGTTGCCATGCAGAAGAGTTCTCGGCGGAGGAATCACCCCCTGGTCACTCAGATGGTGGAAAAATGGCTGGACCGCTACAGGCAGATCCGGCCCTGTACATCCCTGTCTtctagaagttccggatctccgaaaaagaaacgcaaagttggatccggcagcggcgccaccaacttcagcctgctgaagcaggccggcgacgtggaggagaaccccggccccgtgagcaagggcgaggaggataacatggccatcatcaaggagttcatgcgcttcaaggtgcacatggagggctccgtgaacggccacgagttcgagatcgagggcgagggcgagggccgcccctacgagggcacccagaccgccaagctgaaggtgaccaagggtggccccctgcccttcgcctgggacatcctgtcccctcagttcatgtacggctccaaggcctacgtgaagcaccccgccgacatccccgactacttgaagctgtccttccccgagggcttcaaatgggagcgcgtgatgaacttcgaggacggcggcgtggtgaccgtgacccaggactcctccctgcaggacggcgagttcatctacaaggtgaagctgcgcggcaccaacttcccctccgacggccccgtaatgcagaagaagaccatgggctgggaggcctcctccgagcggatgtaccccgaggacggcgccctgaagggcgagatcaagcagaggctgaagctgaaggacggcggccactacgacgctgaggtcaagaccacctacaaggccaagaagcccgtgcagctgcccggcgcctacaacgtcaacatcaagttggacatcacctcccacaacgaggactacaccatcgtggaacagtacgaacgcgccgagggccgccactccaccggcggcatggacgagctgtacaagTAGGCTAGCGTTTAAACTTAAGCTTGGTACCGAGCTCGGATCCACTAGTCCAGTGTGGTGGAATTCTGCAGATATCCAGCACAGTGGCGGCCGCTCGAGTCTAGAGGGCCCGTTTAAACCCGCTGATCAGCCTCGACTGTGCCTTCTAGTTGCCAGCCATCTGTTGTTTGCCCCTCCCCCGTGCCTTCCTTGACCCTGGAAGGTGCCACTCCCACTGTCCTTTCCTAATAAAATGAGGAAATTGCATCGCATTGTCTGAGTAGGTGTCATTCTATTCTGGGGGGTGGGGTGGGGCAGGACAGCAAGGGGGAGGATTGGGAAGACAATAGCAGGCATGCTGGGGATGCGGTGGGCTCTATGGCTTCTGAGGCGGAAAGAACCAGCTGGGGCTCTAGGGGGTATCCCCACGCGCCCTGTAGCGGCGCATTAAGCGCGGCGGGTGTGGTGGTTACGCGCAGCGTGACCGCTACACTTGCCAGCGCCCTAGCGCCCGCTCCTTTCGCTTTCTTCCCTTCCTTTCTCGCCACGTTCGCCGGCTTTCCCCGTCAAGCTCTAAATCGGGGGCTCCCTTTAGGGTTCCGATTTAGTGCTTTACGGCACCTCGACCCCAAAAAACTTGATTAGGGTGATGGTTCACGTAGTGGGCCATCGCCCTGATAGACGGTTTTTCGCCCTTTGACGTTGGAGTCCACGTTCTTTAATAGTGGACTCTTGTTCCAAACTGGAACAACACTCAACCCTATCTCGGTCTATTCTTTTGATTTATAAGGGATTTTGCCGATTTCGGCCTATTGGTTAAAAAATGAGCTGATTTAACAAAAATTTAACGCGAATTAATTCTGTGGAATGTGTGTCAGTTAGGGTGTGGAAAGTCCCCAGGCTCCCCAGCAGGCAGAAGTATGCAAAGCATGCATCTCAATTAGTCAGCAACCAGGTGTGGAAAGTCCCCAGGCTCCCCAGCAGGCAGAAGTATGCAAAGCATGCATCTCAATTAGTCAGCAACCATAGTCCCGCCCCTAACTCCGCCCATCCCGCCCCTAACTCCGCCCAGTTCCGCCCATTCTCCGCCCCATGGCTGACTAATTTTTTTTATTTATGCAGAGGCCGAGGCCGCCTCTGCCTCTGAGCTATTCCAGAAGTAGTGAGGAGGCTTTTTTGGAGGCCTAGGCTTTTGCAAAAAGCTCCCGGGAGCTTGTATATCCATTTTCGGATCTGATCAAGAGACAGGATGAGGATCGTTTCGCATGATTGAACAAGATGGATTGCACGCAGGTTCTCCGGCCGCTTGGGTGGAGAGGCTATTCGGCTATGACTGGGCACAACAGACAATCGGCTGCTCTGATGCCGCCGTGTTCCGGCTGTCAGCGCAGGGGCGCCCGGTTCTTTTTGTCAAGACCGACCTGTCCGGTGCCCTGAATGAACTGCAGGACGAGGCAGCGCGGCTATCGTGGCTGGCCACGACGGGCGTTCCTTGCGCAGCTGTGCTCGACGTTGTCACTGAAGCGGGAAGGGACTGGCTGCTATTGGGCGAAGTGCCGGGGCAGGATCTCCTGTCATCTCACCTTGCTCCTGCCGAGAAAGTATCCATCATGGCTGATGCAATGCGGCGGCTGCATACGCTTGATCCGGCTACCTGCCCATTCGACCACCAAGCGAAACATCGCATCGAGCGAGCACGTACTCGGATGGAAGCCGGTCTTGTCGATCAGGATGATCTGGACGAAGAGCATCAGGGGCTCGCGCCAGCCGAACTGTTCGCCAGGCTCAAGGCGCGCATGCCCGACGGCGAGGATCTCGTCGTGACCCATGGCGATGCCTGCTTGCCGAATATCATGGTGGAAAATGGCCGCTTTTCTGGATTCATCGACTGTGGCCGGCTGGGTGTGGCGGACCGCTATCAGGACATAGCGTTGGCTACCCGTGATATTGCTGAAGAGCTTGGCGGCGAATGGGCTGACCGCTTCCTCGTGCTTTACGGTATCGCCGCTCCCGATTCGCAGCGCATCGCCTTCTATCGCCTTCTTGACGAGTTCTTCTGAGCGGGACTCTGGGGTTCGAAATGACCGACCAAGCGACGCCCAACCTGCCATCACGAGATTTCGATTCCACCGCCGCCTTCTATGAAAGGTTGGGCTTCGGAATCGTTTTCCGGGACGCCGGCTGGATGATCCTCCAGCGCGGGGATCTCATGCTGGAGTTCTTCGCCCACCCCAACTTGTTTATTGCAGCTTATAATGGTTACAAATAAAGCAATAGCATCACAAATTTCACAAATAAAGCATTTTTTTCACTGCATTCTAGTTGTGGTTTGTCCAAACTCATCAATGTATCTTATCATGTCTGTATACCGTCGACCTCTAGCTAGAGCTTGGCGTAATCATGGTCATAGCTGTTTCCTGTGTGAAATTGTTATCCGCTCACAATTCCACACAACATACGAGCCGGAAGCATAAAGTGTAAAGCCTGGGGTGCCTAATGAGTGAGCTAACTCACATTAATTGCGTTGCGCTCACTGCCCGCTTTCCAGTCGGGAAACCTGTCGTGCCAGCTGCATTAATGAATCGGCCAACGCGCGGGGAGAGGCGGTTTGCGTATTGGGCGCTCTTCCGCTTCCTCGCTCACTGACTCGCTGCGCTCGGTCGTTCGGCTGCGGCGAGCGGTATCAGCTCACTCAAAGGCGGTAATACGGTTATCCACAGAATCAGGGGATAACGCAGGAAAGAACATGTGAGCAAAAGGCCAGCAAAAGGCCAGGAACCGTAAAAAGGCCGCGTTGCTGGCGTTTTTCCATAGGCTCCGCCCCCCTGACGAGCATCACAAAAATCGACGCTCAAGTCAGAGGTGGCGAAACCCGACAGGACTATAAAGATACCAGGCGTTTCCCCCTGGAAGCTCCCTCGTGCGCTCTCCTGTTCCGACCCTGCCGCTTACCGGATACCTGTCCGCCTTTCTCCCTTCGGGAAGCGTGGCGCTTTCTCATAGCTCACGCTGTAGGTATCTCAGTTCGGTGTAGGTCGTTCGCTCCAAGCTGGGCTGTGTGCACGAACCCCCCGTTCAGCCCGACCGCTGCGCCTTATCCGGTAACTATCGTCTTGAGTCCAACCCGGTAAGACACGACTTATCGCCACTGGCAGCAGCCACTGGTAACAGGATTAGCAGAGCGAGGTATGTAGGCGGTGCTACAGAGTTCTTGAAGTGGTGGCCTAACTACGGCTACACTAGAAGAACAGTATTTGGTATCTGCGCTCTGCTGAAGCCAGTTACCTTCGGAAAAAGAGTTGGTAGCTCTTGATCCGGCAAACAAACCACCGCTGGTAGCGGTGGTTTTTTTGTTTGCAAGCAGCAGATTACGCGCAGAAAAAAAGGATCTCAAGAAGATCCTTTGATCTTTTCTACGGGGTCTGACGCTCAGTGGAACGAAAACTCACGTTAAGGGATTTTGGTCATGAGATTATCAAAAAGGATCTTCACCTAGATCCTTTTAAATTAAAAATGAAGTTTTAAATCAATCTAAAGTATATATGAGTAAACTTGGTCTGACAGTTACCAATGCTTAATCAGTGAGGCACCTATCTCAGCGATCTGTCTATTTCGTTCATCCATAGTTGCCTGACTCCCCGTCGTGTAGATAACTACGATACGGGAGGGCTTACCATCTGGCCCCAGTGCTGCAATGATACCGCGAGACCCACGCTCACCGGCTCCAGATTTATCAGCAATAAACCAGCCAGCCGGAAGGGCCGAGCGCAGAAGTGGTCCTGCAACTTTATCCGCCTCCATCCAGTCTATTAATTGTTGCCGGGAAGCTAGAGTAAGTAGTTCGCCAGTTAATAGTTTGCGCAACGTTGTTGCCATTGCTACAGGCATCGTGGTGTCACGCTCGTCGTTTGGTATGGCTTCATTCAGCTCCGGTTCCCAACGATCAAGGCGAGTTACATGATCCCCCATGTTGTGCAAAAAAGCGGTTAGCTCCTTCGGTCCTCCGATCGTTGTCAGAAGTAAGTTGGCCGCAGTGTTATCACTCATGGTTATGGCAGCACTGCATAATTCTCTTACTGTCATGCCATCCGTAAGATGCTTTTCTGTGACTGGTGAGTACTCAACCAAGTCATTCTGAGAATAGTGTATGCGGCGACCGAGTTGCTCTTGCCCGGCGTCAATACGGGATAATACCGCGCCACATAGCAGAACTTTAAAAGTGCTCATCATTGGAAAACGTTCTTCGGGGCGAAAACTCTCAAGGATCTTACCGCTGTTGAGATCCAGTTCGATGTAACCCACTCGTGCACCCAACTGATCTTCAGCATCTTTTACTTTCACCAGCGTTTCTGGGTGAGCAAAAACAGGAAGGCAAAATGCCGCAAAAAAGGGAATAAGGGCGACACGGAAATGTTGAATACTCATACTCTTCCTTTTTCAATATTATTGAAGCATTTATCAGGGTTATTGTCTCATGAGCGGATACATATTTGAATGTATTTAGAAAAATAAACAAATAGGGGTTCCGCGCACATTTCCCCGAAAAGTGCCACCTGACGTC |

**Table S3.** Nucleic acid sequences for the construction of SMC1A K1222R cell line

| **Note** | **Sequence** |
| --- | --- |
| gRNA sequence in plasmid | ATTTTTCTTTTTCTCCCTCCTCCTTCAGCAAGGGGACTGTGTGATCAGCAAAGTCCTGACCTTCGACCTCACCAAGTACCCAGATGCCAACCCCAACCCCAATGAGCAGTAGCAGTATTT |
| gRNA 2 | TGGCATCTGGGTACTTGGTGAGG |
| Oligo for homology-directed repair | tctccctcctccttcagCAAGGGGACTGTGTGATCAGCAAAGTCCTGACCTTCGATCTCACCAGGTACCCAGATGCCAACCCCAACCCCAATGAGCAGTAGCAGTATTTTTGCCCTCCCGC |
| WT sequence | actagacccctcttaattctctccttacaattttatttttctttttctccctcctccttcagCAAGGGGACTGTGTGATCAGCAAAGTCCTGACCTTCGACCTCACCAAGTACCCAGATGCCAACCCCAACCCCAATGAGCAGTAGCAGTATTTTTGCCCTCCCGCCCTGTCTGGATCCCTAAGCTGTCCCTCTCCCAATCTCTGGATATTTGACTCCCAA |
| After homology-directed repair sequence: | actagacccctcttaattctctccttacaattttatttttctttttctccctcctccttcagCAAGGGGACTGTGTGATCAGCAAAGTCCTGACCTTCGATCTCACCAGGTACCCAGATGCCAACCCCAACCCCAATGAGCAGTAGCAGTATTTTTGCCCTCCCGCCCTGTCTGGATCCCTAAGCTGTCCCTCTCCCAATCTCTGGATATTTGACTCCCAA |

**Table S4.** RT-qPCR primers used in this work

| **Oligonucleotide** | **Sequence** |
| --- | --- |
| GAPDH-F | GGAGCGAGATCCCTCCAAAAT |
| GAPDH -R | GGCTGTTGTCATACTTCTCATGG |
| SERTAD1-F | CTGGCTGTCTACTGGACGATG |
| SERTAD1-R | TGGTGCCCAAAGTTCATTGTC |
| NUDT16L1-F | TTCGTGAGCACGGCTAAGTG |
| NUDT16L1-R | GGGCATCATGTTGAGCACC |
